# The deficiency of poly-β-1,6-*N*-acetyl-glucosamine deacetylase trigger *A. baumannii* to convert to biofilm-independent colistin-tolerant cells

**DOI:** 10.1038/s41598-023-30065-5

**Published:** 2023-02-16

**Authors:** Shu-Jung Lai, I-Fan Tu, Tien-Sheng Tseng, Yu-Hsuan Tsai, Shih-Hsiung Wu

**Affiliations:** 1 Graduate Institute of Biomedical Sciences, China Medical University, Taichung, 404333 Taiwan; 2 Research Center for Cancer Biology, China Medical University, Taichung, 404333 Taiwan; 3 Institute of Biological Chemistry, Academia Sinica, Taipei, 11529 Taiwan; 4 Institute of Molecular Biology, National Chung Hsing University, Taichung, Taiwan; 5 Institute of Molecular Physiology, Shenzhen Bay Laboratory, Shenzhen, 518132 China; 6 Department of Chemistry, National Taiwan University, Taipei, 106 Taiwan

**Keywords:** Pathogenesis, Antimicrobials, Biofilms, Pathogens

## Abstract

*Acinetobacter baumannii* is a nosocomial pathogen that can be resistant to antibiotics by rapidly modulating its anti-drug mechanisms. The multidrug-resistant *A. baumannii* has been considered one of the most threatening pathogens to our society. Biofilm formation and persistent cells within the biofilm matrix are recognized as intractable problems, especially in hospital-acquired infections. Poly-β-1,6-*N*-acetyl-glucosamine (PNAG) is one of the important building blocks in *A. baumannii*’s biofilm. Here, we discover a protein phosphoryl-regulation on PNAG deacetylase, AbPgaB1, in which residue Ser411 was phosphorylated. The phosphoryl-regulation on AbPgaB1 modulates the product turnover rate in which deacetylated PNAG is produced and reflected in biofilm production. We further uncovered the PgaB deficient *A. baumannii* strain shows the lowest level of biofilm production but has a high minimal inhibition concentration to antibiotic colistin and tetracycline. Based on bactericidal post-antibiotic effects and time-dependent killing assays with antibacterial drugs, we claim that the PgaB-deficient *A. baumannii* converts to colistin-tolerant cells. This study utilizes a biofilm-independent colistin-tolerant model of *A. baumannii* to further investigate its characteristics and mechanisms to better understand clinical outcomes.

## Introduction

In recent decades, increasing antibiotic resistance has resulted in a higher risk of nosocomial pathogenic infections. Carbapenem-resistant *Acinetobacter baumannii* is a common cause of often life-threatening opportunistic infections in critically ill patients. The expression of carbapenemase is the most important mechanism to confer carbapenem resistance in drug-resistant pathogens^1,2^. Evidence such as the loss of the outer-membrane porin^3,4^, differential expression of penicillin-binding protein^5^, and the overproduction of efflux systems^6^ have also been described as involved in carbapenem resistance in *A. baumannii*. Biofilm formation allows *A. baumannii* to colonize in different environments and it is usually associated with virulence^7,8^. The first step to initiating biofilm formation is that planktonic cells need to attach to biotic or abiotic surfaces. Through cell–cell adhesion and cell proliferation procedures, biofilm structures mature and resume a planktonic lifestyle when it disperses to new environments. Several factors associated with biofilm formation in *A. baumannii* have been identified, including the CsuA/BABCDE pili usher-chaperone assembly system^9,10^, the BfmS/BfmR two-component system^11^, outer membrane protein OmpA^12,13^, biofilm-associated protein^14,15^, autoinducer synthase AbaI^16^, and the protein complex PgaABCD that is required for poly-beta-1–6-*N*-acetylglucosamine (PNAG) synthesis^17^.

Partially deacetylated PNAG (dPNAG) is considered a required exopolysaccharide to structure biofilms in several human pathogens, such as *A. baumannii*^17^, *Aggregatibacter* spp.^18^, *Bordetella pertussis*^19,20^, *Klebsiella pneumonia*^21^, *Staphylococcus aureus*^22^, and *S. epidermidis*^23^. Exopolysaccharide dPNAG is polymerized and translocated via the PgaABCD system in *A. baumannii*^17^. The homologous system in *E. coli* shows that PgaC and PgaD are required for PNAG polymerization^24^. The polymerized PNAG is partially deacetylated by PgaB and subsequently translocated out by PgaA^24^. The biofilm detection results of the *E. coli* knockout strains revealed that PgaA and PgaB are necessary for PNAG translocation while the PgaC deletion strain possessed undetectable polymerized PNAG^17,24^. PNAG transporter PgaA possessed several negatively charged residues inside its β-barrel secretion pore for initial binding to dPNAG^25^. The site-directed mutation and biofilm detection revealed that the negatively charged residues in the PgaA secretion pore result in the preference to interact with positive-charged dPNAG^25^. Therefore, fully acetylated PNAG was not considered to be exported out to serve as a biofilm-supporting exopolysaccharide^25^.

The PNAG *N*-deacetylase PgaB plays a critical role in the regulation of acetyl levels of PNAG and serves as a bridge for dPNAG translocation by PgaA^26^. PgaB’s N- and C-terminal domains are both required to process PNAG de-*N*-acetylation^26^. The catalytic region of de-*N*-acetylation is located at PgaB’s N-terminal domain while the C-terminal domain possesses glycosyl hydrolysis activity and it is critical for PNAG exportation^20,26^. Based on crystal structure analysis of EcPgaB, a specific β-hairpin loop (residues 610–623) was claimed to interact with PNAG/dPNAG for export^26^. PgaB showed cobalt- and nickel-dependent activity to partially deacetylate β-1,6-glucosamine but not β-1,4-glucosamine oligomer^27^. The specific de-*N*-acetylation position on glucosamine pentasaccharide occurred at the 2nd or 3rd monosaccharide from the nonreducing terminus^27^. Although the de-*N*-acetylation positions were determined using fully acetylated β-1,6-glucosamine pentasaccharide coupled with the treatment of exoglycosidase SpHex^27^, the natural exopolysaccharide PNAG isolated from bacteria still displayed high diversity of length and its deacetylated positions.

The glycosyl hydrolase activity of *Bordetella bronchiseptica* PgaB (BbPgaB) discovers a new modulating mechanism in PNAG production, demonstrating the glycoside digestion of deacetylated PNAG^20^. The C-terminal domain of BbPgaB was categorized into glycoside hydrolase family 153 (GH153) and orthologous to *E. coli* PgaB^20^. The deacetylation activity of PgaB requires both N- and C-terminal domains, while the truncated C-terminal domains of BbPgaB and EcPgaB can hydrolyze dPNAG^20^. The required motif of dPNAG polymer for cleavage was identified as GlcN-GlcNAc-GlcNAc which demonstrated that fully acetylated PNAG was not recognized as a substrate for the C-terminal domain of BbPgaB^20^.

Biofilm matrices are considered physical protectants for bacteria, leading to higher antibiotic tolerance. Proteomic and mutagenesis studies demonstrated that OmpA, Omp33, CarO, OprD-like protein, putative DcaP-like protein, and histidine metabolism are essential to biofilm formation^21^. However, there is less information on studying drug resistance in *A. baumannii* to link to its PNAG-mediated biofilm formation. Previous comparisons in the transcriptome of *A. baumannii* clinical strains revealed that the transcriptional level of PgaB (A1S_0938) in a colistin-resistant strain was significantly higher than in a colistin-susceptible strain^28^. Protein post-translational modifications are reversible regulations that modulate protein functions in response to several physiological reactions. Our previous study uncovered that PgaB from *A. baumannii* clinical strain SK17 was phosphorylated^29^. This study confirmed the phospho-regulation of PgaB-mediated biofilm production in *A. baumannii* ATCC15151. According to the site-directed mutation and *N*-deacetylation activity assay, phospho-modification on residue Ser411 of PgaB showed a significantly higher turnover rate that resulted in higher production of dPNAG serves as the building block for biofilm. We noticed that the amount of PNAG-mediated biofilm production was negatively correlated to colistin resistance in *A. baumannii*. The PgaB deletion strain produced the lowest amount of biofilm but possessed significantly higher colistin resistance. We hypothesize that the PgaB-deficient *A. baumannii* strain accumulates PNAG at the periplasm which may impede colistin’s ability to target the cytoplasmic membrane of *A. baumannii*.

## Results

### Sequence analysis of poly-*N*-acetyl-glucosamine synthesis operon in *A. baumannii*

**Figure 1:**
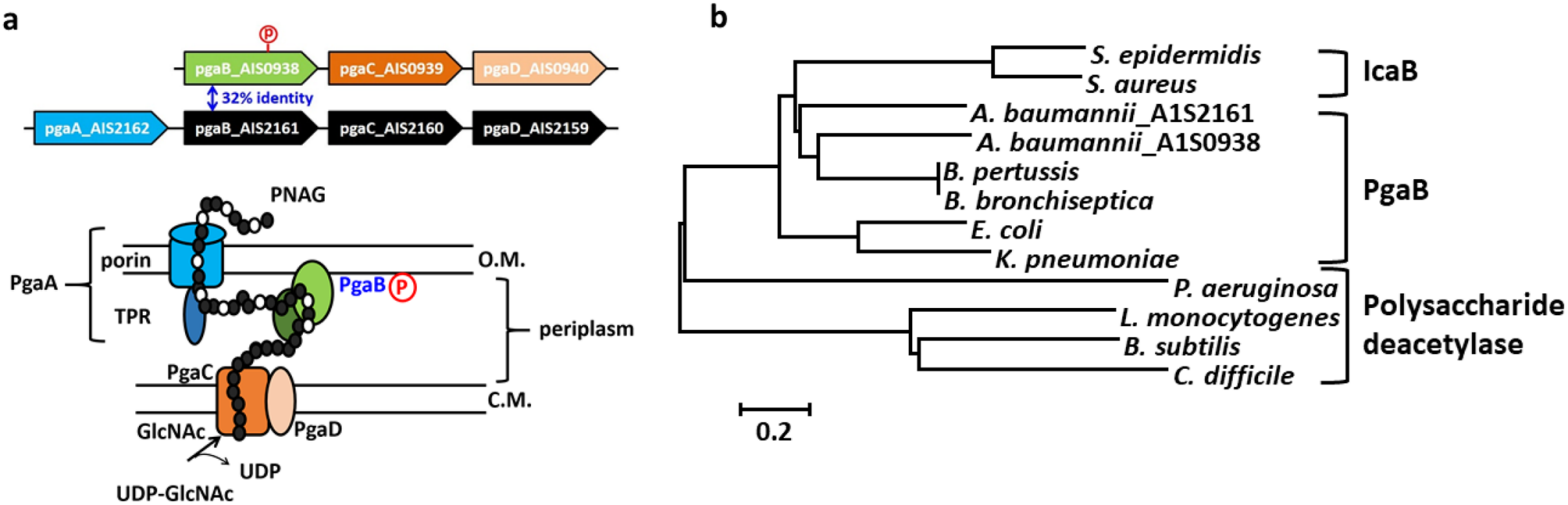
Schematic illustration of two PNAG synthesis operons in *A. baumannii*. (**a**) The coding genes A1S-0938 to A1S-0940 and A1S-2162 to A1S-2159 were annotated as PNAG synthesis operons in *A. baumannii*. PgaC (brown) and PgaD (orange) Protein complexes were located on the cytoplasmic membrane to polymerize *N*-acetylglucosamine. PgaB is a PNAG deacetylase that anchors on the outer membrane and is located at the periplasm. Outer membrane transporter protein PgaA contributed to the transport of partially deacetylated PNAG as an exopolysaccharide. (**b**) Phylogenetic analysis of polysaccharide deacetylases in CE4 family of CAZy database from bacteria based on Neighbor Joining clustering method.

In Pga operons, *N*-acetylglucosamine deacetylase PgaB possesses the ability to regulate the degree of acetylation on PNAG which directly modulates the binding affinity of PNAG to the PgaA β-barrel lumen structure^25^. Partial deacetylated PNAG (dPNAG) was then transported out as an extracellular polysaccharide via PgaA (Fig. 1a). The TPR domain of PgaA in *E. coli* participated in the protein–protein interaction to PgaB which is critical for partially deacetylated PNAG exportation^30^. Based on this information, we hypothesized that biofilm formation in *A. baumannii* may be regulated by *N*-deacetylase PgaB. There are two Pga operons, A1S0938 to A1S0940 and A1S2162 to A1S2159, among all sequenced *A. baumannii* strains (Fig. 1a). Both copies of *N*-acetylglucosamine deacetylase (A1S0938 and A1S2161) were annotated as PgaB and were categorized into carbohydrate esterase family (CE4 family) within the currently defined Carbohydrate-Active Enzyme database (CAZy). The coding proteins of genes A1S0938 and A1S2161 were defined respectively as AbPgaB1 and AbPgaB2 in this study. PgaB is an outer-membrane-anchored protein in which residues S12 to N194 of AbPgaB1 and H35 to W70 of AbPgaB2 are transmembrane regions predicted by TMRPres2D^31^ (Fig. S1). AbPgaB1 shares 35.68% and 41.64% sequence identities to EcPgaB and BbPgaB, respectively. According to phylogenetic analysis, AbPgaB1 (A1S0938) is close to *B. bronchiseptica* which may have glycoside hydrolysis activity at the C-terminal domain (Fig. 1b). Although AbPgaB1 and AbPgaB2 share low sequence identity (32%), they still are clustered together within PgaB from *Escherichia coli*, *Klebsiella pneumonia*, *B. bronchiseptica*, *B. pertussis* and IcaB from *Staphylococcus* but not polysaccharide deacetylase from *Pseudomonas*. (Fig. 1b).

PgaB is considered to modulate the ratio of acetyl-group on PNAG. However, the regulation of PgaB to produce differential acetylated PNAG as a biofilm matrix is still unclear. Single-gene deletion (Δ*AbpgaB1*) and double-gene deletion (Δ*AbpgaB1*Δ*AbpgaB2*) strains of *A. baumannii* were constructed for exopolysaccharide analysis. Acetyl-level exopolysaccharide (including PNAG) extracted from *A. baumannii* ATCC 15151 (Ab15151) and its derivative mutant strains were determined based on proton-NMR analysis (Fig. S2). The signal of acetylated PNAG was relatively quantified based on the integral area of peak at 2.0 ppm on proton-NMR profiles (Fig. S2). The acetylation level of extracted polysaccharides was slightly increased from Δ*AbpgaB1* and it was further increased in the double deletion. The extracted exopolysaccharide from the *pgaB* double deletion strain represented higher acetyl-level signals than Ab15151 which demonstrated the *N*-deacetylase activity of both AbPgaB1 and AbPgaB2 could modulate the acetylation level of PNAG in Ab15151.

### PNAG *N*-deacetylase AbPgaB1 was phosphoryl-modified in *A. baumannii*

**Figure 2:**
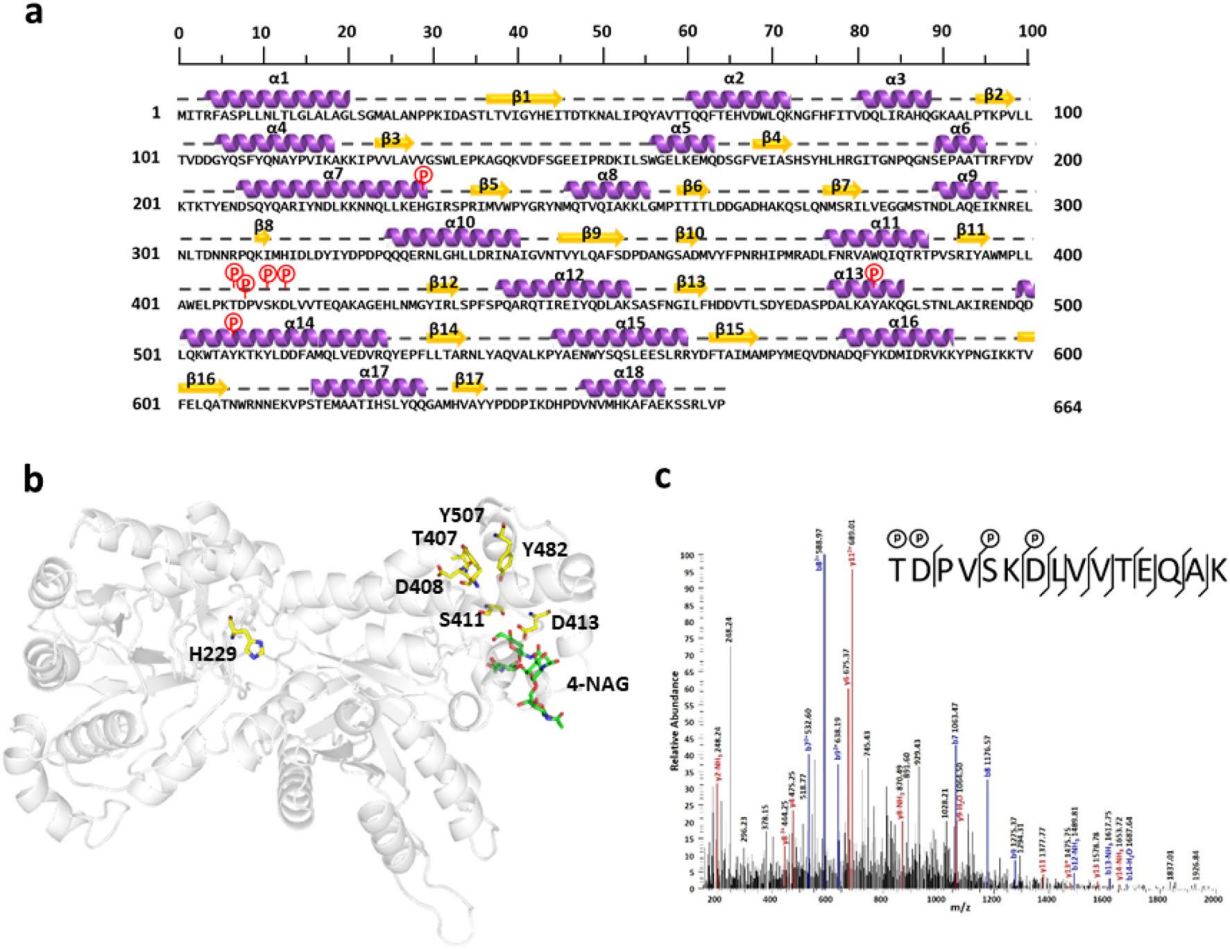
The identified phosphorylated sites on AbPgaB1 marked in secondary structure and tertiary structure. (**a**) The secondary structure of AbPgaB1 was predicted by Jpred4. Alpha helix and beta sheet structures were marked in purple and yellow, respectively. Identified p-sites were marked “p” (red) on AbPgaB1 sequences. (**b**) Tetrasaccharide of PNAG, 4-NAG, which the main chain showed in sticks (green) were docked into the AbPgaB1 modeled structure (cartoon, predicted based on template PDB: 4P7R^26^ by SWISS-MODEL) to display the location of phosphorylated residues (yellow) within the 3D structure. The modeled structure was edited using PyMOL. (**c**) MS/MS spectra of p-peptide “^407^TDPVSKDLVVTEQAK^421^” on AbPgaB1. Each peak reveals the m/z of fragments after tandem mass spectrometry separation and is processed by MaxQuant. The N-terminal b-ion and C-terminal y-ion fragments were highlighted in blue and red, respectively.

We performed both proteomic and phosphoproteomic analyses to investigate the differential expressed proteins between planktonic and biofilm lifestyles of *A. baumannii*. With a combination of proteomic data, 1334 constitutive expressed proteins could be identified from both planktonic and biofilm lifestyles of Ab15151 (Fig. S3). There were 174 and 226 unique proteins that were only identified from the planktonic and biofilm of *A. baumannii*, respectively (Fig. S3). Among these identified proteins, AbPgaB1 (A1S0938) was defined as a reliable phosphoprotein via phosphoproteomic analysis using the standard procedures described in Methods. The coding gene of AbPgaB1 was constructed and transformed into Ab15151 for overexpression and its phosphorylated sites (p-sites) were confirmed based on purified AbPgaB1 through LC–MS/MS analysis. Seven unambiguous p-sites from four phosphorylated peptides (p-peptides) on AbPgaB1 were identified (Table S1). According to the protein’s secondary structure prediction of AbPgaB1 by Jpred4^32^, p-site H229 is located at α-helix 7 (α7) in the N-terminal domain while the p-sites T407, D408, S411, and D413 are located at loop 11 in C-terminal domain (Fig. 2a). There were two other p-sites, Y482 and Y507, located at α13 and α14 in C-terminal domain, respectively (Fig. 2a). The structure of AbPgaB1 was predicted by SWISS-MODEL based on *E. coli* PgaB crystal structure as a template (PDB: 4P7R)^26^. According to the MS/MS data, seven p-sites on AbPgaB1 were marked on the N- and C-domains which were respectively annotated to have PNAG deacetylase activity and glycosyl hydrolysis activity (Fig. 2b). To figure out the regulation of phosphoryl-modification on AbPgaB1, the tetrasaccharide of *N*-acetylglucosamine (4-NAG) was docked into AbPgaB1 modeled structure (Fig. 2b). The p-sites S411 and D413 were close to 4-NAG in AbPgaB1 which revealed that they may be candidates for the investigation of the phosphoryl-regulation on AbPgaB1. The MS/MS spectra of p-peptide “^407^TDPVSKDLVVTEQAK^421^” in loop 11 which contained p-sites T407, D408, S411, and D413 are shown in Fig. 2c. These results demonstrate that the phosphoryl-regulation of the C-terminal domain of AbPgaB1, specifically residues S411 and D413, may modulate its activity.

### AbPgaB1 *N*-deacetylase activity is phosphoryl-regulated at residue Ser411 and modulates the biofilm production

According to the modeling structure analysis, we hypothesized that p-site Ser411 was critical in binding and releasing product dPNAG. Residue Ser411 of AbPgaB1 was then replaced by Ala or Asp to mimetic non-phosphorylated or phosphorylated conditions. The AbPgaB1 and its site-direct-mutated derivatives were respectively constructed into the pABCLIIa expression vector to generate C-terminal His-tag fusion proteins for further affinity purification. Overexpressed AbPgaB1 and its derivatives were purified for deacetylation activity assay by using partial deacetylated PNAG as substrate. Kinetic parameters of AbPgaB1 deacetylase activity revealed that the *K*_*m*_ value was increased by 4.9 times when Ser411 was replaced with Asp (Table 1). On the other hand, the maximum velocity of AbPgaB1 was enhanced by 8.4 times when Ser411 was phosphorylated (Table 1). In comparison to non-phosphorylated S411A mutated AbPgaB1, phospho-mimetic AbPgaB1 (S411D) possessed a dramatically higher turnover number, implying more deacetylated PNAG production.

**Table 1:** Comparison of the kinetic parameters between AbPgaB1 and its Ser411-mediated mutants.

	*K*_*m*_ (mg mL^−1^)	*V*_*max*_ (μg mL^−1^·min^−1^)	*K*_*cat*_ (min^−1^)	*K*_*cat*_·*K*_*m*_^−1^
AbPgaB1_WT	4.80 ± 0.06	0.92 ± 0.04	0.0184 ± 0.0008	0.0038 ± 0.0002
AbPgaB1_S411A	4.09 ± 0.01	0.23 ± 0.02	0.0045 ± 0.0004	0.0011 ± 0.0001
AbPgaB1_S411D	23.32 ± 2.15	7.72 ± 1.49	0.1544 ± 0.0297	0.0066 ± 0.0007

To figure out the phosphoryl-mediated regulation of AbPgaB1, the modeling structure of the AbPgaB1 C-terminal domain (residues 308–659, AbPgaB1_308–659_) was built based on GlcNAc tetrasaccharide incorporated EcPgaB (PDB: 4P7R)^26^ crystal structure as a template. Modeling structure AbPgaB1_308–659_ was aligned to EcPgaB (PDB: 4P7R) and *B. bronchiseptica* PgaB (BbPgaB, PDB: 6AU1)^20^ which showed highly conserved GlcNAc tetrasaccharide interacting residues (Fig. S4a,b). According to the crystal structure analysis of EcPgaB, residue W552 forms a tightly stacking interaction with the pyranoid ring and D472 hydrogen bonds to OH-3 and OH-4 of GlcNAc. Spatial rearrangements of residues W549 and D470 from AbPgaB1 and residues W561 and D480 from BbPgaB were highly conserved to residues W552 and D472 in EcPgaB, implying the participation of these residues in GlcNAc tetrasaccharide binding (Fig. S4a). In addition, residue D473 of AbPgaB1_308–659_ was identical to residue D475 in EcPgaB (PDB: 4F9J) which was considered to form bidentate hydrogen bonds to GlcNAc tetrasaccharide^27^. Therefore, residues nearby, such as W549, D470, and D473 in AbPgaB1_308–659_ were defined as the active sites allowing flexible docking of the GlcNAc tetrasaccharide ligand. Among the distinct rotamers resulting from this interaction, the one with the lowest energy was selected as the GlcNAc tetrasaccharide incorporated into the modeled structure of AbPgaB1_308–659_ (Fig. S4c).

The reducing end of GlcNAc tetrasaccharide in the AbPgaB1_308–659_ modeling structure was defined as the + 1 subunit (Fig. S4c). In AbPgaB1_308–659_, residue W549 participates in the stacking interaction with the pyranoid ring and its main-chain hydrogen bonding to the *N*-acetyl moiety of + 2 GlcNAc. The oxygen of the main chain on residue Y430 formed a hydrogen bond *N*-acetyl moiety of the + 1 subunit of GlcNAc (Fig. S4c). AbPgaB1_308–659_ was strongly coordinated to + 1 GlcNAc by a hydrogen bonding network, including the side-chain of residues D413, R432, E472, S469, T467, and the main chain of Y430. The single CH–π interaction between residue E472 and + 1 GlcNAc also contributed to ligand binding (Fig. S4c). The conserved R432-E472 salt-bridge (Fig. S4b) may contribute to + 1 GlcNAc accommodation and stabilize those loops in PNAG binding (Fig. S4b,c). Therefore, phosphorylated mimic Ser411 (S411D) located near R432 would break the pre-existing R432-E472 salt bridge to trigger a partial unfolding of the loops^27^ (salt-bridge competition model)^33^ (Fig. S4d,e). This event would likely affect the hydrogen bonding network allowing + 1 GlcNAc accommodation. Moreover, compared to the modeled wild-type complex, the bulk phosphoryl group of S411D in AbPgaB_308–659_ would cause the spatial repulsion of PNAG to hinder its binding (Fig. S4d,e).

**Figure 3:**
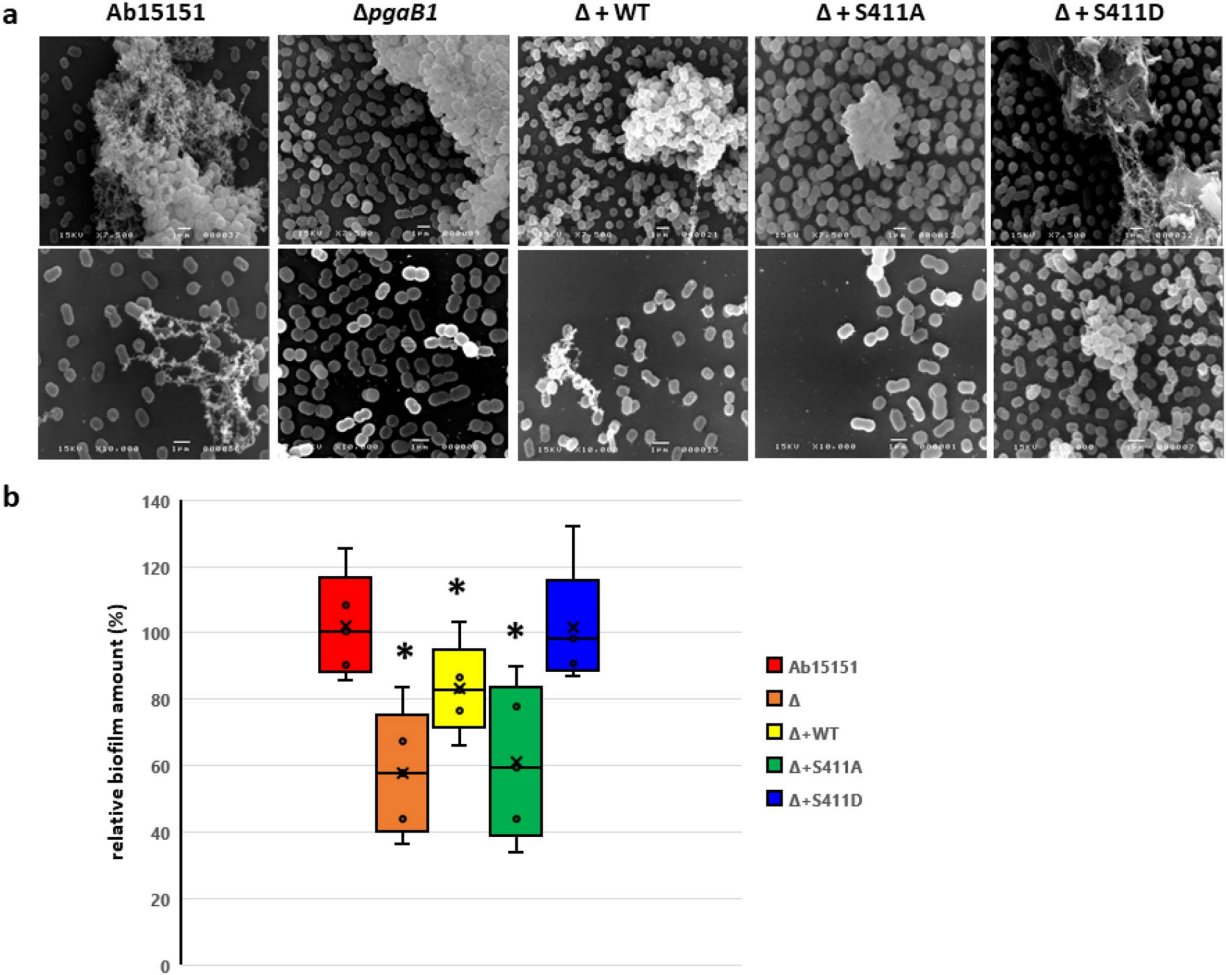
Observation and quantification of biofilm production in *A. baumannii* ATCC15151 and the *AbpgaB1*-mediated mutant strains. (**a**) Observation of biofilm formation in Ab15151 and its derivatives via SEM. The upper panel showed the view with 7,500 × magnification and the lower panel showed with 10,000 × magnification. The bar indicated a scale of 1 µm. (**b**) Biofilm was quantified by crystal violet staining and determined the absorbance at 595 nm. The OD_595_ values of Ab15151 were defined as 100% to calculate the relative biofilm amounts from its derivative mutant strains. Each data point was averaged from at least 6 repeats. *Indicated a significant difference from Ab15151 whose p-value of the t-test was less than 0.001.

To investigate the effects of phospho-mediated regulation on AbPgaB1 in biofilm formation, Ab15151 and its derivative mutant strains were observed by scanning electron microscope (SEM). Biofilms from wild-type Ab15151 showed aggregated structure and cell-attached exopolysaccharide (Fig. 3a). AbPgaB1 deletion strain (Δ*AbpgaB1*) displayed aggregated cells in biofilm matrix without observable exopolysaccharide (Fig. 3a). This cell-attached exopolysaccharide was produced when *AbpgaB1* was complimented. Furthermore, we noticed that the complemented phospho-mimetic AbPgaB1 (Δ + S411D) showed both cell aggregation and cell-attached exopolysaccharide in biofilm structure. Conversely, there was no cell-attached exopolysaccharide observed from the strain complemented with non-phospho-mimetic AbPgaB1 (Δ + S411A) (Fig. 3a). The amount of biofilm production from Ab15151 and its derivative mutant strains were quantified by crystal violet staining. All the quantified values were normalized based on the biofilm amounts from Ab15151. *A. baumannii* produce less biofilm (58%) when its *AbpgaB1* was knockout (Fig. 3b). Biofilm production was recovered to 83% of Ab15151 when wild-type *AbpgaB1* was complemented to *AbpgaB1* deletion strain (Fig. 3b). It is noteworthy that the relative biofilm amounts from strain complemented with non-phosphorylated AbPgaB1 (Δ + S411A) and phosphorylated AbPgaB1 (Δ + S411D) were 61% and 101%, respectively (Fig. 3b). It demonstrated that PNAG deacetylation level was regulated by phosphoryl-mediated modification at residue Ser411 on AbPgaB1, reflected biofilm production in *A. baumannii.*

### PgaB deficient strain had biofilm-independent antibiotic tolerance to colistin and tetracycline

The biofilm production ability in *A. baumannii* trended in a positive direction relative to its toxicity and drug resistance. The above evidence reveals that biofilm production in *A. baumannii* is regulated by one residue phosphoryl-modification on AbPgaB1. We further hypothesized that the antibiotic resistance in *A. baumannii* should decrease when AbPgaB1 is dephosphorylated at residue Ser411. Since *A. baumannii* has two copies of *pgaB* in the genome, we constructed single and double *pgaB* deletion strains for further antibiotic susceptible assays. According to the minimal inhibitory concentration (MIC) determination based on the broth dilution method, there is no significant difference in MIC values against imipenem, ciprofloxacin, apramycin, and vancomycin among tested *A. baumannii* strains (Table 2). It revealed the independence of biofilm formation and the tolerance to these four antibiotics. We noticed that MIC values of *A. baumannii* strains to colistin and tetracycline were significantly increased (colistin 2.0 to 16.0 mg/L and tetracycline 0.5 to 16.0 mg/L) when both *AbpgaB1* and *AbpgaB2* were deleted (Table 2, Δ*AbpgaB1*Δ*AbpgaB2*, ΔΔ). These data disprove our hypothesis that the PgaB double-deletion-strain produced the lowest amounts of biofilm among *A. baumannii* strains in this study, but possessed the highest tolerance to antibiotic colistin and tetracycline (Table 2, Fig. S5). Colistin, also known as polymyxin E, could target lipopolysaccharide (LPS) in bacteria to disrupt their cell membrane integrity to kill bacteria. It is considered a last-resort treatment for infection by multidrug-resistant pathogens. To the best of our knowledge, there is no evidence to support the correlation between PNAG deacetylase PgaB and the structure of LPS. Interestingly, the tolerance of *A. baumannii* against colistin is related to its PNAG *N*-deacetylase PgaB. And there is evidence that colistin affects LPS in the cytoplasmic membrane^34^. Therefore, we further hypothesized that the PgaB double-deletion strain displayed a PNAG pump-out deficient strain in which acetylated PNAG accumulates at periplasm to impede the disruption by colistin treatment. However, we still need more evidence to figure out the detailed mechanism involved in colistin tolerance when both PgaBs were deleted.

**Table 2:** Minimal inhibition concentration to Ab15151 and its derivative mutant strains.

MIC (µg/mL)	Colistin	Imipenem	Ciprofloxacin	Apramycin	Vancomycin	Tetracycline
Ab15151	1.0–2.0	0.25–0.5	0.13–0.25	4.0–8.0	62.5–125.0	0.5–1.0
Δ*pgaB1*	1.0–2.0	0.25–0.5	0.13–0.25	4.0–8.0	62.5–125.0	0.5–1.0
Δ*pgaB1*Δ*pgaB2* (ΔΔ)	2.0–16.0*	0.5–1.0	0.13–0.25	4.0–8.0	62.5–125.0	0.5–16.0*
ΔΔ + *pgaB1*	1.0–4.0	0.5–1.0	0.13–0.25	4.0–8.0	62.5–125.0	0.25–0.5
ΔΔ + *pgaB1*_S411A	1.0–2.0-	0.5–1.0	0.13–0.25	2.0–4.0	62.5–125.0	0.25–0.5
ΔΔ + *pgaB1*_S411D	1.0–2.0	0.25–1.0	0.13–0.25	4.0–8.0	62.5–125.0	0.5–1.0
P. aeruginosa**	2.0–4.0	2.0–4.0	0.25–1.0	4.0–8.0	> 500	8.0–16.0

*During 4 times repeat MIC determination, the tolerance to antibiotics was increased.

**MIC to *P. aeruginosa* ATCC 27853 was performed as standard control.

Tetracycline is a kind of antibiotic to bind 30S ribosomes and results in the inhibition of protein synthesis. PgaB double deletion strains had higher tetracycline MIC values (Table 2). Apramycin is another antibiotic used in this study. Apramycin targets protein synthesis to inhibit bacteria. The MIC to apramycin showed no difference among all tested *A. baumannii* strains in this study (Table 2). Therefore, we considered that the tetracycline tolerance of PgaB double deletion strains is caused by its deficient ability to penetrate bacteria cells. However, more studies are needed to validate this hypothesis. According to the MIC of *AbpgaB1* complemented Ab strains, either WT, S411A, or S411D mutated *Ab*PgaB1, in Δ*AbpgaB1* or Δ*AbpgaB1*Δ*AbpgaB2* background showed similar susceptibility to colistin and tetracycline (Table 2, Fig. S6). This indicated that colistin or tetracycline tolerance occurred in both PgaB deletion strains and that it could be recovered by *AbpgaB1* complementation (Table 2).

To figure out the antibiotic tolerability in biofilm conditions when *AbpgaB1* and/or *AbpgaB2* are deleted, the minimum biofilm eradication concentrations (MBEC) were examined (Table 3). The MBEC of all tested strains is higher than the MIC indicating that the bacterial cells embedded in the biofilm can overcome higher antibiotic stresses. There is no significantly different MBEC of Δ*AbpgaB1*Δ*AbpgaB2* strain and the other tested strains. The MBEC data reveals that the colistin and tetracycline tolerance in Δ*AbpgaB1*Δ*AbpgaB2* strain was not contributed by the biofilm production. To confirm the drug tolerance that occurred in the PgaB double deletion strain was repeatable, the MIC of antibacterial drugs was determined in 4 passages. We noticed that the MIC of Δ*AbpgaB1*Δ*AbpgaB2* strain to colistin and tetracycline was increased when it was sub-transferred from the 1^st^ passage to the 4^th^ passage (Fig. S6). This phenomenon was repeatable in at least three independent tests. It prompted us to propose the hypothesis that PgaB-deficient *A. baumannii* can convert to antibiotic tolerant cells to overcome the bactericidal effect of colistin. The persistent cells show a population of bacteria that survive exposure to a bactericidal antibiotic^35^. Antibiotic persistent bacteria do not result in a MIC increase but are killed at a lower rate than non-persistent cells^35^. The increased MIC of Δ*AbpgaB1*Δ*AbpgaB2* strain to colistin and tetracycline during 4-passages indicated that it is not contributed by the amounts of persistent cells. Starvation stress is the major selective pressure during 4-passage incubation. It demonstrated that the deficiency of PgaB in *A. baumannii* may lead to the evolution to overcome antibiotic stresses.

**Table 3:** Minimum biofilm eradication concentration to Ab15151 and its derivative mutant strains.

MBEC (µg/mL)	Colistin	Imipenem	Ciprofloxacin	Apramycin	Vancomycin	Tetracycline
Ab15151	62.5–125.0	31.3–62.5	2.0–3.9	125.0–250.0	> 500.0	31.3–62.5
Δ*pgaB1*	62.5–125.0	62.5–125.0	7.8–15.6	250.0–500.0	> 500.0	31.3–62.5
Δ*pgaB1*Δ*pgaB2* (ΔΔ)	31.3–125.0	62.5–500.0	7.8–15.6	250.0–500.0	> 500.0	62.5–125.0
ΔΔ + *pgaB1*	62.5–125.0	125.0–500.0	7.8–15.6	62.5–500.0	> 500.0	31.3–62.5
ΔΔ + *pgaB1*_S411A	62.5–125.0	125.0–500.0	7.8–15.6	62.5–500.0	> 500.0	31.3–62.5
ΔΔ + *pgaB1*_S411D	31.3–125.0	125.0–500.0	7.8–15.6	250.0–500.0	> 500.0	62.5–125.0
P. aeruginosa*	62.5–125.0	> 500.0	7.8–15.6	250.0–500.0	> 500.0	250.0–500.0

*MBEC to *P. aeruginosa* ATCC 27853 was performed as standard control.

**Figure 4:**
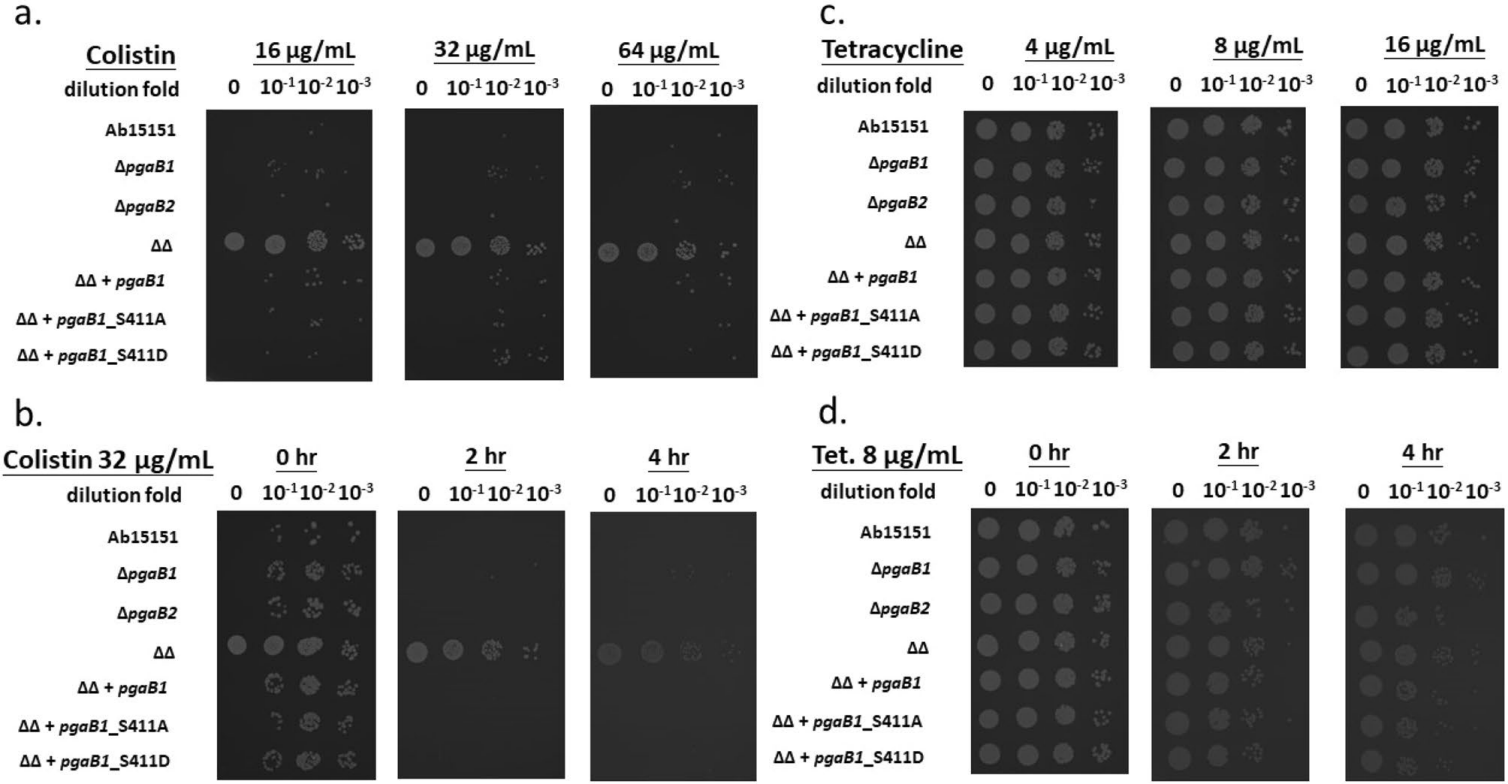
Time-dependent post-antibiotic effects of colistin and tetracycline to *A. baumannii* strains in this study. The overnight cultures were diluted to OD_600_ 0.1 for colistin or 0.01 for tetracycline administration. After a 1 h treatment with colistin (**a**) or tetracycline (**c**), the cultures were tenfold serially diluted to spot on the LB agar plate. With the administration of 32 µg/mL colistin (**b**) or 8 µg/mL tetracycline (**d**) within 4 h, all tested strains were tenfold-diluted and incubated overnight for evaluating their survival rate.

Post-antibiotic effects (PAE) of colistin on *A. baumannii* were performed to investigate the antibiotic tolerance among WT and mutant strains in this study. The overnight cultured Ab strains were diluted to OD_600_ 0.1 for colistin administration. After one hour of colistin treatment with tested concentration, each tested Ab strains were serial diluted and spotted on LB agar plates (Fig. 4a). We noticed that Ab15151, Δ*AbpgaB1*, and Δ*AbpgaB2* were all susceptible to colistin in the concentration of 16.0–64.0 µg/mL (Fig. 4a). However, the PgaB double deletion strain (Δ*AbpgaB1*Δ*AbpgaB2*) showed the highest tolerance when treated with 16.0, 32.0, or 64.0 µg/mL of colistin for 1 h (Fig. 4a). To distinguish antibiotic persistence from tolerance, Δ*AbpgaB1*Δ*AbpgaB2* strain was killed in a slower rate than other strains in presence of 16.0 µg/mL colistin for 0 to 4 h treatment (Fig. 4b). The susceptibility of PgaB double deletion strain was recovered when complemented with *AbpgaB1* (Table 2, Fig. 4a,b). Both dose-dependent and time-dependent PAE demonstrated that the *pgaB*-double deletion strain possesses high colistin tolerance. It supports our hypothesis that post colistin treatment, the persistent cells of PgaB-deficient *A. baumannii* may have the ability to convert to colistin-tolerant cells. It demonstrates the incidence of evolution occurring in the Δ*pgaB1*Δ*pgaB2* strain may be faster than the other tested *A. baumannii* strains in this study. The tetracycline PAE showed similar profiles among Ab strains in all tested doses and periods. (Fig. 4c,d). It revealed that the initial loaded bacterial cells (~ 10^6^ CFU/mL) were not killed by the bacteriostatic drug, tetracycline, in the concentration of 4.0, 8.0, or 16.0 µg/mL treatment for 1 h (Fig. 4c) or after 8.0 µg/mL tetracycline administration for 4 h (Fig. 4d). We noticed that the antibiotic persistence in the PgaB double deletion strain was not observed when administration with bactericidal drug ciprofloxacin or the bacteriostatic drug apramycin (Fig. S7). This means the PgaB deficient *A. baumannii* is specifically resistant to antibiotic colistin and tetracycline.

**Figure 5:**
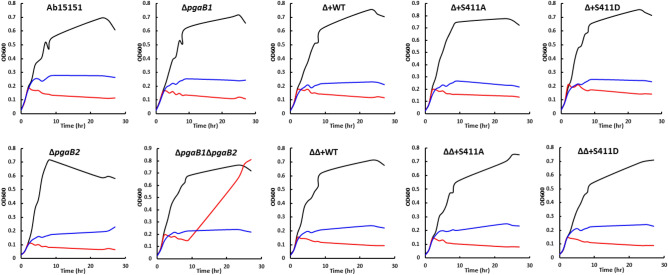
Time-dependent killing assays of WT and PgaB-mediated *A. baumannii* mutant strains demonstrated its colistin tolerance. In this study, the overnight cultures of *A. baumannii* strains were diluted to OD_600_ 0.05 for growth curve determination. The antibiotic colistin (red) and tetracycline (blue) were administrated with the final concentration of 16.0 µg/mL after 2 h incubation. During 27 h incubation, the optical density of each culture was determined at 600 nm by a microplate reader. The growth curve drawn in black was the condition without antibiotic administration as a control.

The specific characteristics of the antibiotic persistent cells are the replication rate in the presence of antibiotics was lower than in non-persister cells^35^. To distinguish the antibiotic-tolerant cells from hetero-resistant cells, the time-dependent killing assays of *A. baumannii* strains under antibiotic administration were carried out. All tested *A. baumannii* strains showed a similar growth curve without antibiotic administration which revealed the *pgaB* deletion and complement strains in this study did not affect the replication (Fig. 5). The cultures of Ab strains with 16.0 µg/mL tetracycline showed stagnant growth among all tested strains (Fig. 5). The growth curves of all tested Ab strains that were administrated 16.0 µg/mL colistin were also stagnant except for PgaB deficient strain (Δ*AbpgaB1*Δ*AbpgaB2*) (Fig. 5). The replication of Δ*AbpgaB1*Δ*AbpgaB2* strain was also inhibited when treated with colistin, however, the growth was recovered after a period of lag time (Fig. 5). We hypothesize that the survived cells in PgaB deficient background may convert to colistin-tolerant cells and grew-up during overnight incubation.

## Discussion

This study's construction of *pgaB*-mutant strains was based on its homologous recombination region to knockout *AbpgaB1* or *AbpgaB2* on the Ab15151 genome. To rule out the polar mutation effects on downstream *pgaC* and *pgaD* expression, the *pgaBCD* (A1S0938 ~ A1S0940) were constructed into expression vector pABCLIIb and then transform into *pgaB*-deleted Ab15151 (Δ*AbpgaB1* or Δ*AbpgaB1*Δ*AbpgaB2*) as *AbpgaB1* complement strains (+ WT, + S411A, or + S411D). The biofilm formation assays showed that the *AbpgaB1* complement strain (Δ + WT) can recover the biofilm production (Fig. 3b). The MIC determination also indicated that the *AbpgaB1* complement strains (including S411A or S411D mutated *AbpgaB1*) could recover susceptibility to colistin and tetracycline in Δ*AbpgaB1*Δ*AbpgaB2* background (Fig. S4). According to our previous experiences, the complemented protein induced by IPTG in broth dilution MIC determination may not be constitutively expressed^36^. We also confirmed the PgaB1 expression in broth dilution MIC assays by using Western to detect His-tag fusion signals on complemented AbPgaB1 (Fig. S8). The complemented AbPgaB1 could recover biofilm production in Δ*AbpgaB1* background and recover the susceptibility to both colistin and tetracycline in Δ*AbpgaB1*Δ*AbpgaB2* background. Our data supports that there were no polar mutation effects in our constructed Ab strains.

In this study, we use ninhydrin to quantify the free amine exposed when PNAG was deacetylated by AbPgaB1. The substrate of AbPgaB1 is PNAG isolated from *A. baumannii* clinical strain SK17. Since both PGA operons exist in all sequenced *A. baumannii* strains, PNAG should be one of the components in their exopolysaccharides. The specific de-*N*-acetylation position was uncovered by using fully-acetylated pentasaccharide PNAG as substrate^27,37^. The native PNAG isolated from bacteria still displayed a high diversity of acetylation levels, deacetylated positions, and molecular weights. To figure out the phosphoryl-regulation on residue Ser411 in AbPgaB1, the kinetic parameters between AbPgaB1 and its site-direct mutant derivatives were determined using ninhydrin assays with native extracted PNAG as substrate. We harvested one batch of native-extracted PNAG for all of the ninhydrin activity assays in this study to limit the variation between different batches of native-extracted PNAG. Due to the extracted PNAG already being partially deacetylated, the specific activity of AbPgaB1 in this study may not compare to other published data at the same baseline. However, it is appropriate to compare the kinetic parameters between AbPgaB1 and its mutant in this study by using PNAG isolated from the same batch. Based on activity assays of AbPgaB1 and site-directed mutant S411A or S411D, we conclude that phosphorylated AbPgaB1 (S411D) has a higher product dPNAG turnover number than WT and nonphosphorylated AbPgaB1 (S411A) (Table 1). It reveals that AbPgaB1_S411D has a higher efficacy to deacetylate PNAG for PgaA pump-out as a biofilm building block. This result is also consistent with biofilm quantification and SEM observation among Ab15151 and its derivative strains (Fig. 3).

To the best of our knowledge, there are two PGA operons highly conserved in all sequenced *A. baumannii* strains. Protein post-translational phosphorylation was found in PNAG *N*-deacetylase AbPgaB1 (A1S_0938) rather than AbPgaB2 (A1S_2161). Individual AbPgaB1 or AbPgaB2 deletion strains (Δ*AbpgaB1* or Δ*AbpgaB2*) and the PgaB double deletion strain (Δ*AbpgaB1*Δ*AbpgaB2*) were constructed to elucidate the contribution for biofilm production and antibacterial drugs tolerances. The AbPgaB1 deletion Ab15151 strain (Δ*AbpgaB1*) produces less biofilm, of which phenotype was reversed in Δ*AbpgaB1* complemented with *AbpgaB1*_WT (Δ + WT) or *AbpgaB1* contained phospho-mimetic mutant on residue Ser411 (Δ + S411D) (Fig. 3). High acetyl level of PNAG appears to bind to PgaA with a less extent against exopolysaccharide export^30^, reflecting the less biofilm production in Δ*AbpgaB1*Δ*AbpgaB2* (Fig.S5). The PgaB deficient strain (Δ*AbpgaB1*Δ*AbpgaB2*) possessed high tolerance to colistin and tetracycline among all tested strains (Table 2 and Fig. 4). This result demonstrated a biofilm-independent strategy of *A. baumannii* to overcome antibiotic stresses. The previous study mentioned that PgaB deficient *E. coli* is not able to form biofilm due to PNAG accumulated at periplasm^24^. The study of BbPgaB in *B. bronchiseptica* showed that the BbPgaB and its deacetylation activity are not required for the PNAG translocation^38^. There are two possibilities to result in the BbPgaB-independent PNAG translocation in *B. bronchiseptica*^38^. One is the smaller TPR domain in BbPgaA than EcPgaA, which possess the ability to interact with PgaB to affect its PNAG translocation activity. It was confirmed by further study of protein–protein interaction between the TPR domain of PgaA and PgaB^30^. The other possibility is the deficient residues in the BbPgaA TPR domain may lead to the porin structure constitutive open for PNAG translocation^38^. The sequence alignment of AbPgaA (A1S-2162, 812 a.a.) and EcPgaA (807 a.a.) shows 55% of sequence identity which indicated the highly conserved TPR domain and porin structure between AbPgaA and EcPgaA. The AbPgaA modeling structure which builds by SWISS-MODEL with PDB 4y25 as a template shows that the porin structure of AbPgaA possesses several negatively charged residues (Fig. S9). This structural characteristic is consistent with the hypothesis described in the EcPgaA study that AbPgaA may prefer to translocate deacetylated PNAG than acetylated PNAG. We considered that the PgaB deficient strain (Δ*AbpgaB1*Δ*AbpgaB2*) in this study may accumulate PNAG in the periplasm hindering colistin to neutralize LPS on the cytoplasmic membrane and result in higher colistin tolerance (Table 2 and Fig. 4).

Due to the nephrotoxicity and neurotoxicity of colistin, the use of this antibacterial drug is getting rare. However, colistin is still considered a last-resort antibiotic against gram-negative bacteria, especially in carbapenem-resistant MDR pathogen infection. Recent reports reassessed the risk of colistin’s side effects and demonstrate its safety and high efficiency against MDR gram-negative pathogen infection^39–42^. The rise of isolated clinical carbapenem-resistant MDR pathogens makes colistin a last-resort antibiotic to be considered. Currently, there are more and more heteroresistant and therapeutic failures in colistin treatment which means the colistin-resistant mechanisms that need to be identified^43–45^. The current studies of colistin resistance are involved in chromosomally mediated and plasmid-mediated research. The loss of genomic regions containing *mrkC*, *mrkD*, *modA*, *modB*, *modC*, *modD*, and *ppk*, which participate in biofilm production was observed in colistin-resistant *A. baumannii* strains^46^. The *mcr-1* and *mcr-2* genes that mediate plasmid-borne colistin resistance were reported^47,48^. The two-component systems PmrA/PmrB and PhoP/PhoQ are associated with the regulation of LPS modification^40,42^. These reports demonstrated that most colistin-resistant mechanism focuses on LPS modification and pili-assembly-mediated biofilm production. In this study, we discovered a new possibility of *A. baumannii* overcoming colistin by hindering PNAG transportation as the biofilm building block. And somehow this phenotype of *A. baumannii* could convert to colistin-tolerant cells that survive when stimulated with the bactericidal antibiotic colistin.

In conclusion, the PNAG-associated biofilm production was regulated by PNAG deacetylase, PgaB, which generally existed in *A. baumannii*. There are two copies of PgaB involved in *A. baumannii*’s biofilm production. AbPgaB1 is phosphoryl-regulated on residue Ser411 to modulate the dPNAG turnover number and is directly associated with biofilm production. Both MIC determination and antibacterial drug-killing assays revealed that the PgaB deficient *A. baumannii* strain (Δ*AbpgaB1*Δ*AbpgaB2*) has a higher tolerance to the antibiotics colistin and tetracycline than wild-type and other derivative strains in this study. Depending on the MIC determination during 4 passages, we considered that PgaB deficient *A. baumannii* strain (Δ*AbpgaB1*Δ*AbpgaB2*) was triggered to convert to colistin-tolerant cells easier than others during subculture for four passages without any antibacterial drug administration. This study discovers a new phenotypic model to figure out the transition of colistin-tolerant *A. baumannii.* Because the production of PNAG may differ among different *A. baumannii* strains and sometime it may not be contributed to increase the level of biofilm production among all of the clinical strains. This study could provide a possible mechanism to figure out the colistin-resistance of *A. baumannii* in clinical.

## Materials and methods

### Sequence alignment and phylogenetic analysis

The sequences of A1S-0938 and A1S-2161 were downloaded from National Center for Biotechnology Information (NCBI, https://www.ncbi.nlm.nih.gov/) and their accession numbers were ABO11370 and ABO12588, respectively. Sequence identity was analyzed by Needleman-Wunsch alignment on NCBI. Polysaccharide deacetylase from bacteria was aligned by ClustalW and the phylogenetic tree was drawn based on the Neighbor-Joining clustering method by using Molecular Evolutionary Genetics Analysis version 6.0 (MEGA6)^49^. The other accession numbers of protein sequences used in this study are as follows: *pgaB* from *E. coli* (AWY89024), *K. pneumonia* (AUH99166), *B. bronchiseptica* (AUV49813), IcaB from *S. aureus* (AAD52057), *S. epidermidis* (AAZ78359), polysaccharide deacetylase from *P. aeruginosa* (AAG04906), *L. monocytogenes* (NP_463944), *B. subtilis* (API95827), and *C. difficile* (AYD08208). Transmembrane region of A1S-0938 (*AbpgaB1*) and A1S-2161 (*AbpgaB2*) were predicted by the TransMembrane protein Re-Presentation in 2Dimensions’ tool (TMRPres2D)^31^.

### Construction of mutant strains

*A. baumannii* strain 15151 was regularly cultured at 37 °C in LB medium with shaking. To construct the *pgaB* deletion Ab strains, the fragment contained 600 bp upstream of A1S_0938 (including full-length A1S_0937 and the A1S_0938 promoter) and 1300 bp downstream of A1S_0938 (containing full-length A1S_0939) and was defined as *dpgaB1*. The fragment of *dpgaB1* was cloned into the pK18mobsacB vector (ATCC87097™). The constructed plasmid was then transformed into *E. coli* S17-1 for conjugation to *A. baumannii* strain 15151. The constructed *dpgaB1* was integrated into the chromosome based on homologous recombination with a kanamycin-resistant gene as a selection marker. Knockout mutants were selected by growing the cells on a medium containing 10% sucrose without antibiotic treatment. Both *AbpgaB1* and *AbpgaB2* double deletion Ab strains followed a similar strategy to the construction of the *AbpgaB2* (A1S_2161) fragment and the conjugation target was the AbPgaB1-deletion Ab strain (Δ*AbpgaB1*). For in-trans complementation, the fragment harboring the genes A1S_0938, A1S_0939, and A1S_0940 was cloned into the shuttle vector pABCLIIc, derived from pABYM2^50^, to generate *AbpgaB1* complement Ab strain (Δ + WT). Mutants mimicking phosphorylated and non-phosphorylated AbPgaB1 were generated via site-directed mutagenesis using *AbpgaB1* complement strain as the template. For further affinity column purification, *AbpgaB1* WT and its derivative mutants were subcloned into shuttle vector pABCLIIa consisting of LacI, LacO, and 6 × His-tag fusion at the C-terminal of the target.

### Extraction of extracellular polysaccharide and NMR analysis

*A. baumannii* strains (WT, Δ*AbpgaB1*, and Δ*AbpgaB1*Δ*AbpgaB2*) were cultured in an LB medium at 37 °C with shaking. In total 1 L of cultures of each strain was harvested and resuspended in 50 mL of DI water. Crude extracts of extracellular polysaccharide (EPS) were resolved in DI water and then suspended cultures were incubated at 100 °C double-boiled water for 30 min. After cooling down to room temperature, the insoluble matrix was removed by centrifugation with 12,000 rpm for 1 h at 4 °C. Total extracts of EPS were precipitated with a final concentration of 75% of ethanol at 4 °C overnight. The precipitants were harvested by centrifugation at 12,000 rpm for 1 h at 4 °C and were resolved in buffer (25 mM Tris, pH 8.0, 5 mM MgCl_2_, 5 mM CaCl_2_). Samples were treated with DNase (Roche) and RNase (Sigma) at 37 °C for 8 h and then treated with proteinase K (Bioscience) at 37 °C for overnight to remove contaminated nucleic acids and proteins. Total extracts were incubated at 100 °C double-boiled water for 30 min to denature all possible remaining proteins and then centrifuged at 12,000 rpm for 1 h at 4 °C. Supernatants were dialysis into the 100-fold volume of DI water by 1 KDa membrane at 4 °C. The samples were lyophilized for further analysis. To relatively quantify the acetylation level of EPS, the lyophilized EPS was resolved in D_2_O for proton NMR analysis (Bruker UltraShield, 600 MHz/54 mm, 14.1 Tesla superconducting magnet).

### Growth conditions and total protein extraction

*A. baumannii* strain 15151 was regularly cultured at 30 °C at an initial OD_600_ of 0.1 in LB medium for proteome and phosphoproteome sample preparation. The culture was harvested after 6 h when the OD_600_ reached 0.4, defined as the mid-exponential phase of the planktonic cells in this study. Biofilm cells were harvested after 24 h of sub-culture, discarding the liquid component and washing the wall-attached biofilms three times with PBS. The biofilm cells were shaken down by adding PBS in wells and shaking the microplate for 15 min. Total extracts of both planktonic and biofilm cells were obtained by sonication. Protein concentrations were quantified based on the Bradford assay (Bio-Rad).

### Nanoscale liquid chromatography-mass spectrometry (LC–MS/MS)

Five milligrams of the extracted proteins were digested using trypsin (1:40 w/w) in both gel-based and gel-free procedures^51,52^. The tryptic peptides were desalted through SDB-XC StageTip for further proteomic analysis. Phosphopeptides from planktonic and biofilm cells were enriched using custom-made HAMMOCK tips, prepared using 0.5 mg TiO_2_ beads (GL Sciences) packed into 10-μL C8-StageTips^46^. The resulting phosphopeptides were lyophilized for further liquid chromatography-electrospray ionization mass spectrometry (LC–ESI–MS, Fusion) analysis (Thermo Scientific). The MS and MS/MS raw data were analyzed using MaxQuant software (version 1.5.1.2)^50^ based on the database of *A. baumannii* strain 15151^53^. The false-discovery rate of the peptides, proteins and modification sites was set to 0.1% and the minimum MaxQuant score for phosphorylation sites was 40, with a localization probability of at least 75%. The gene ontology of each identified protein was annotated based on the Uniprot database (www.uniprot.org). The MS proteomics and phosphoproteomics data were deposited in the ProteomeXchange Consortium via the PRIDE^54^ partner repository, under the dataset identifiers PXD010140 and PXD010172, respectively.

### Molecular modeling of the PgaB-PNAG complex

The protein structure of AbPgaB1 in this study was modeled by the software SWISS-MODEL with template PDB: 4f9j. The modeling structure was displayed by PyMOL. It was considered that the modeling structure could provide reliable information between ligands and proteins. The sketch molecules and prepare ligand modules implemented in Discovery Studio 3.5 (Accelrys Software, Inc., San Diego, CA, USA) were employed to construct the molecular structures of all compounds. Compounds used in the docking analysis were prepared in three steps: (1) two-dimensional structures were converted into three-dimensional structures, (2) charges were calculated, and (3) H atoms were added. Molecular modeling was used to reproduce the complex structure of the AbPgaB1-PNAG complex. AbPgaB1 residues V410, S411, K412, D413, L414, A422, G423, E424, H425, L426, W427, M428, G429, L431, R432, D444, T467, L468, S469, E472, W549, and Y550 were defined as constituting the binding site in protein–ligand flexible docking, which was achieved using the GOLD docking program (Cambridge Crystallographic Data Center (CCDC), version 5.1) with the GoldScore scoring function. The side chains of the binding site residues were set to be flexible during the docking analysis. The constructed, energy-minimized, *N*-acetylglucosamine tetrasaccharide was docked into the defined binding site according to modified docking parameter settings (number of operations = 1,600,000 and population size = 1000; default settings were used for the other parameters). The most likely orientation and the most favorable free energy position were analyzed.

### AbPgaB1 expression and purification

A single colony of *A. baumannii* 15151 carrying the plasmid allowing the overexpression of AbPgaB1 fused with C-terminal His tag was inoculated in the LB medium. The overnight cultures were diluted into 1 L of fresh LB medium at a ratio of 1:100 (v/v). AbPgaB1 expression was induced by the addition of 0.5 mM IPTG when the A_600_ of the cultures reached 0.7. After 12 h of incubation, the cells were harvested and disrupted using a high-pressure homogenizer (Nanolyzer) to obtain total proteins for further purification on a nickel-charged affinity resin (GE Healthcare) according to standard procedures. The purified proteins were concentrated using an Amicon Ultra centrifugal filter and their concentration was quantified based on a Bradford assay. The purified proteins were separated by SDS-PAGE on a 12% acrylamide gel (Tools, HR gradient gel, TFU-GG420).

### De-*N*-acetylation assays

In a previous study, we showed that the major component of exopolysaccharide from *A. baumannii* strain SK17 was partially deacetylated PNAG (dPNAG), which was extracted as the substrate of AbPgaB1. The de-*N*-acetylation activity of AbPgaB1 was performed by ninhydrin assays^55^. Briefly, the de-*N*-acetylation reaction mixture contained 50 mM NaCl, 10 μM CoCl_2_, and 0 ~ 4.0 mg extracted PNAG/mL, prepared in 50 mM HEPES buffer (pH 7.5). The reaction (100 μL final reaction volume) was initiated by the addition of AbPgaB1 or its derivatives. After a 1-h incubation at 37 °C, the reaction was terminated by a 10-min incubation at 100 °C. Colorization was achieved by incubating 50 µL of supernatant with 25 μL of ninhydrin (Sigma) at 100 °C for 10 min. The mixture was diluted by the addition of 125 μL of 95% ethanol and the amount of free amine (de-*N*-acetylated glucosamine) was detected by measuring the absorbance at 570 nm followed by a comparison with a glucosamine standard curve.

### Biofilm observation and quantification

Overnight cultures were diluted in an LB medium containing 1% glucose to obtain an initial OD_600_ of 0.05. After 12 h of incubation at 30 °C with 180 rpm shaking, the resulting biofilm was washed three times in water. To quantify biofilm formation, biofilms attached to the wells of a polypropylene 96-well plate were stained with crystal violet (CV) for 20 min, washed three times in water, and solubilized with 95% ethanol for 10 min, and their absorbance at 595 nm was determined. For scanning electron microscopy (SEM) observation, biofilms formed on coverslips under the same condition were fixed in a 2.5% formaldehyde/4% glutaraldehyde solution and then dehydrated.

### Minimal inhibition concentration detection

The overnight cultures of *A. baumannii* strains were diluted with fresh Mueller–Hinton broth with an initial optical density of 0.005 at 600 nm. The antibiotics were serially diluted for administration to Ab strains. The inoculated cells with serially diluted antibiotics were incubated at 37 °C for 16 h. After incubation, the optical density at 600 nm of tested cultures was determined by the microplate reader. During several passages of MIC determinations, the overnight (16–20 h) cultured Ab strains were sub-transferred with a 1:1000 ratio in LB medium. The MIC detection of *P. aeruginosa* ATCC 27,853 was performed as the standard control in this test.

### Minimum biofilm eradication concentrations

The minimum biofilm eradication concentrations of *A. baumannii* ATCC15151 and its AbpgaB1/AbpgaB2-mediated mutant strains were determined by protocol^56^. Briefly, the overnight cultures were diluted with Mueller–Hinton broth to OD_600_ 0.005. Each strain was cultured in 96-well plates with peg lid placed into each well. After 24 h incubation, the peg-lids have each strains’ biofilm were then transferred into the 96-well plates with Mueller–Hinton broth containing two-fold-diluted antimicrobial solution. The plates were then incubated for 24 h to stimulate with antibiotics. The peg-lids were then placed into new 96-well plates with fresh Mueller–Hinton broth. The biofilm embedded bacterial cells of each tested strains will be released into fresh medium after 1 min sonication. The 96-well plates were incubated for another 24 h and then the lowest concentration of antibiotics which prevented visible growth was defined as the MBEC.

### Post-antibiotic effects

To investigate the survival cells when *A. baumannii* is treated with the bactericidal drug colistin or bacteriostatic drug tetracycline, the 3rd generation cultures of *A. baumannii* strains were diluted to OD_600_ 0.1 (10^7^ CFU/mL) and OD_600_ 0.01 (10^6^ CFU/mL) for colistin and tetracycline administration, respectively. The cultures of Ab strains were treated with 16.0, 32.0, or 64.0 μg/mL of colistin for 1 h for the dose-dependent colistin-killing assays. In the case of tetracycline, the dose-dependent killing assays were performed with 4.0, 8.0, or 16.0 μg/mL of tetracycline. After 1 h of treatment, the cultures were tenfold serial diluted and then spotted 2 μL on the LB agar plate for another overnight incubation at 37 °C. The time-dependent killing assays were performed with 32.0 μg/mL of colistin or 8.0 μg/mL of tetracycline administration for 4 h. During the antibiotic stimulation, the cultures were tenfold serially diluted at 0, 2, and 4 h time-points and spotted on LB agar plate for incubation overnight.

### Growth test

To distinguish the colistin-tolerant cells from hetero-resistant cells, the 3rd generation of Ab strains was diluted to OD_600_ 0.05 by MHB for growth curve determination. The inoculated *A. baumannii* cells were incubated at 37 °C with shaking for 2 h and then administrated with 16.0 μg/mL of colistin or tetracycline for another 25 h incubation. The growth condition without antibiotics was determined as a control. The optical density at 600 nm of each Ab culture was detected at several time points during the 27 h incubation.

## Supplementary Information

Supplementary Information 1.

Supplementary Information 2.

## Data Availability

The detailed list of the identified phosphoproteins and phosphopeptides in this study was supplied as Table S2. The MS proteomics and phosphoproteomics raw data were deposited in the ProteomeXchange Consortium via the PRIDE partner repository, under the dataset identifiers PXD010140 and PXD010172, respectively. All data generated or analyzed during this study are included in this manuscript and its supplementary information files.
