# Supplementary material for: The deficiency of poly-β-1,6-N-acetyl-glucosamine deacetylase trigger A. baumannii to convert to biofilm-independent colistin-tolerant cells
Source: Sci Rep. 2023 Feb 16;13:2800. doi: 10.1038/s41598-023-30065-5 (PMC9935895; doi:10.1038/s41598-023-30065-5)
Supplement: Supplementary file 1 — Supplementary Information 1. [file 41598_2023_30065_MOESM1_ESM.docx]

**Supplemental information**

**The deficiency of poly-β-1,6-N-acetyl-glucosamine deacetylase trigger *A. baumannii* to convert to biofilm-independent colistin-tolerant cells**

Shu-Jung Lai^1,2^*, I-Fan Tu^3^, Tien-Sheng Tseng^4^, Yu-Hsuan Tsai^5^, and Shih-Hsiung Wu^3,6^*

^1^ Graduate Institute of Biomedical Sciences, China Medical University, Taichung, 404333, Taiwan.

^2^ Research Center for Cancer Biology, China Medical University, Taichung, 404333, Taiwan.

^3^ Institute of Biological Chemistry, Academia Sinica, Taipei 11529, Taiwan.

^4^ Institute of Molecular Biology, National Chung Hsing University, Taichung, Taiwan.

^5^ Institute of Molecular Physiology, Shenzhen Bay Laboratory, Shenzhen 518132, China.

^6^ Department of Chemistry, National Taiwan University, Taipei 106, Taiwan.

*To whom correspondence should be addressed:

Shu-Jung Lai, Graduate Institute of Biomedical Sciences, China Medical University, Taichung, 404333, Taiwan and Research Center for Cancer Biology, China Medical University, Taichung, 404333, Taiwan.

Tel: +886-4-22053366 ext. 6732

E-mail: [sjlai01@gmail.com](mailto:sjlai01@gmail.com)

Shih-Hsiung Wu, Institute of Biological Chemistry, Academia Sinica, Taipei 11529, Taiwan

Fax: +886-2-26539142

Tel: +886-2-27855696 ext. 7101

E-mail: [shwu@gate.sinica.edu.tw](mailto:shwu@gate.sinica.edu.tw)


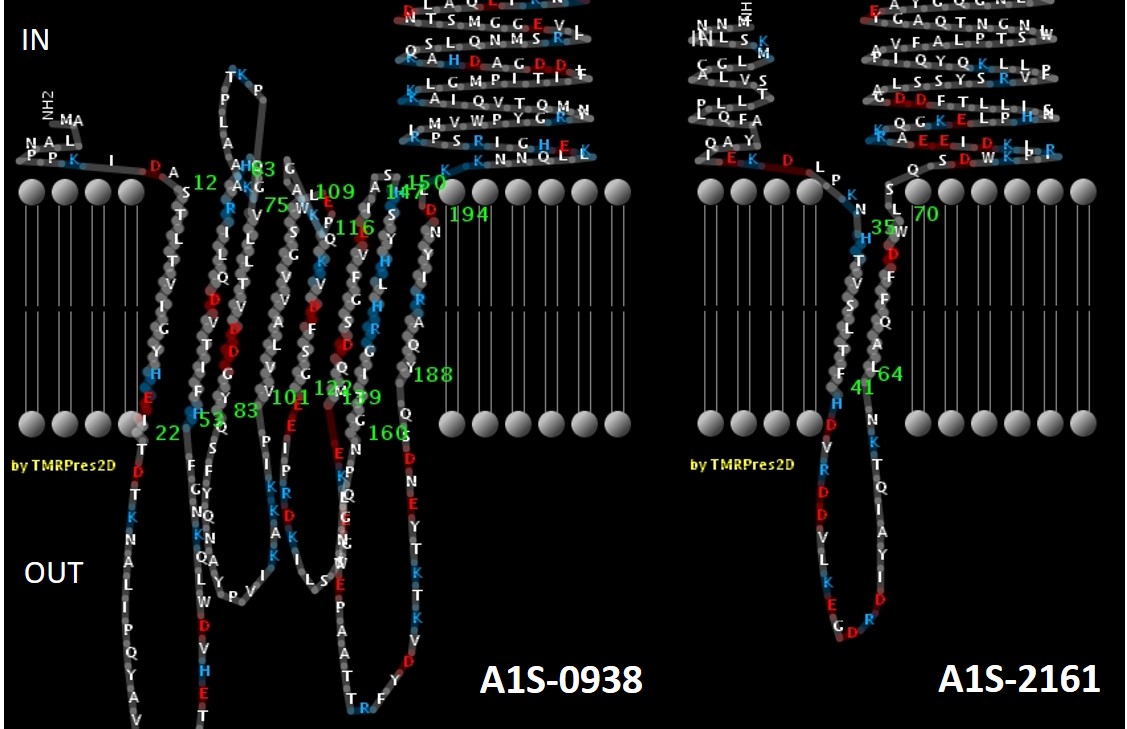


**Fig. S1. Transmembrane regions of PgaB1 and PgaB2 were predicted by TMRPres2D.** Residue S12 to D194 was the transmembrane region of PgaB1 (A1S-0938) while residue H35 to W70 was the transmembrane region of PgaB2 (A1S-2161). IN and OUT indicated inside and outside of outer membrane of bacteria cell.


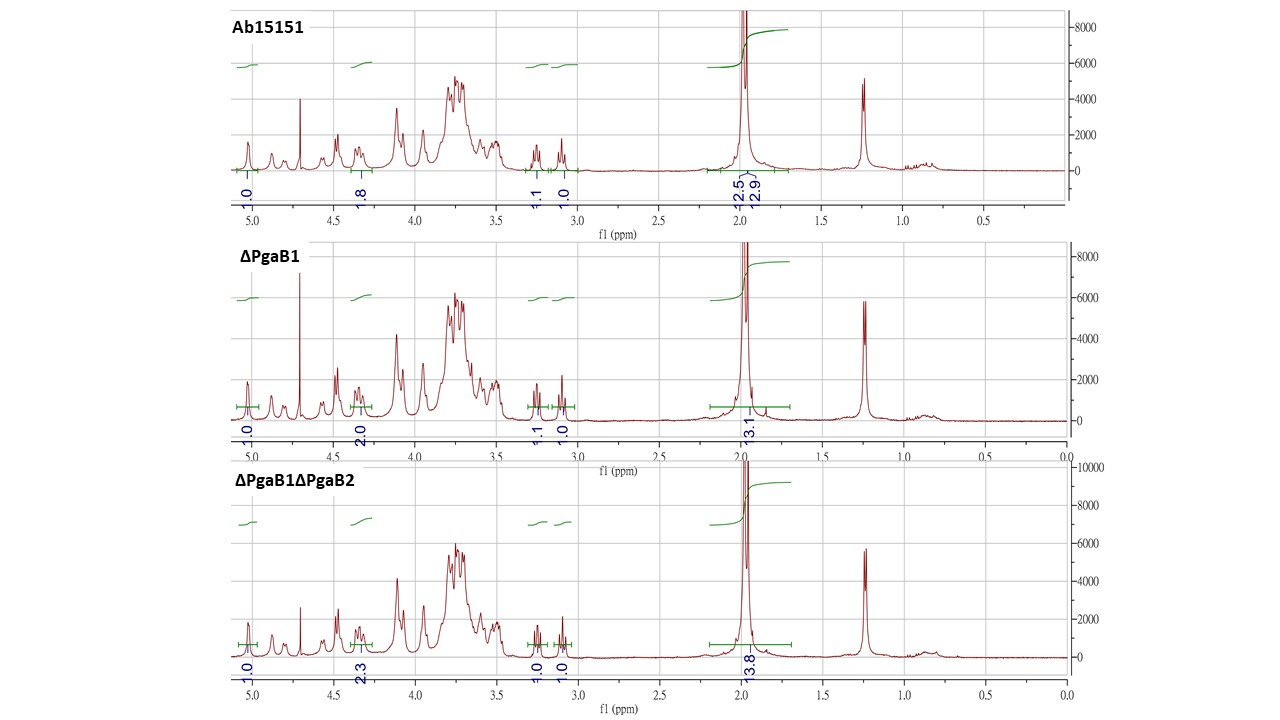


**Fig. S2. PgaB double-deletion strain of *A. baumannii* produces higher acetylated polysaccharide PNAG by proton-NMR determination.** Extracellular polysaccharides from Ab15151, ΔPgaB1, and ΔPgaB1ΔPgaB2 mutant strains were extracted as described in Methods. Brown peaks from 0 to 5.0 ppm showed the proton-NMR spectra. The peaks at 3.1 ppm and 5.0 ppm were used as an internal control to normalize the quantity of each sample. The integral area of peaks at 2.0 ppm was calculated to reveal the differential acetylated level of PNAG among three *A. baumannii* strains.


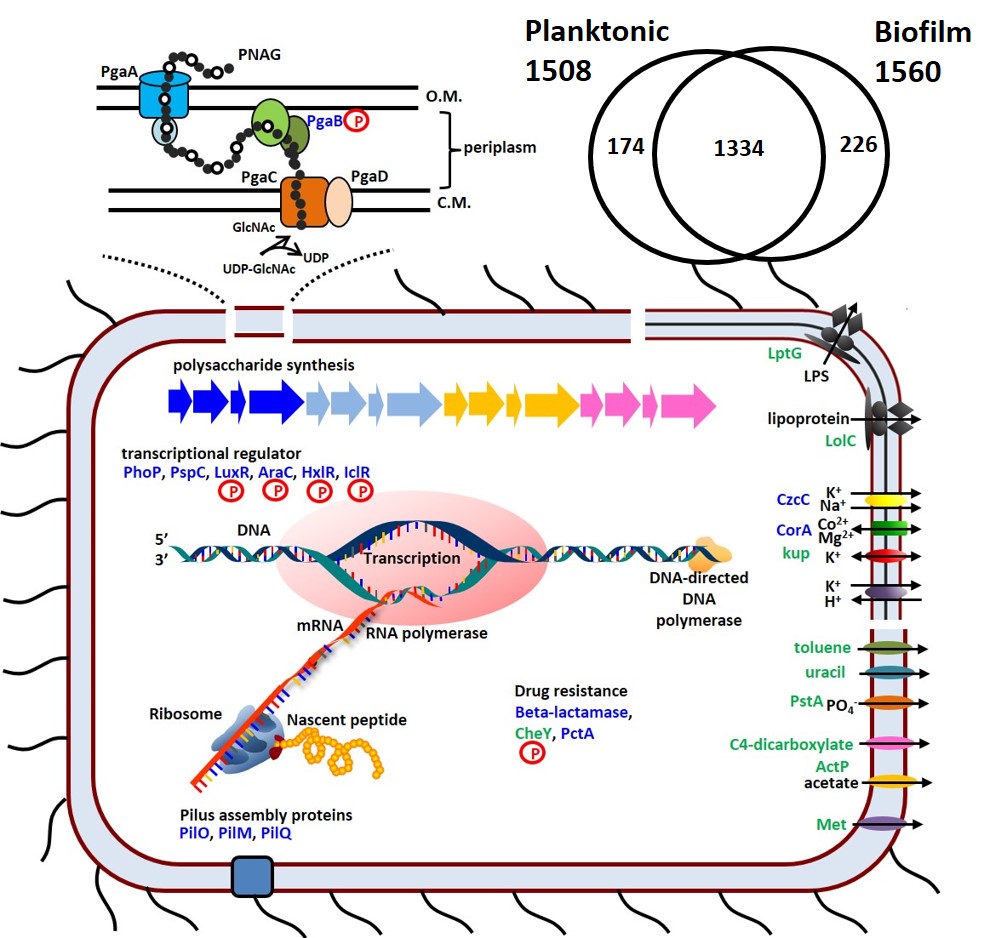


**Fig. S3. Schematic illustration of unique expressed proteins between planktonic and biofilm lifestyles of *A. baumannii* ATCC 15151.** The unique proteins identified from planktonic cells were marked in blue while the unique proteins from biofilm cells were highlighted in green. The PgaA, PgaC, and PgaD which involved in PNAG synthesis are identified from both planktonic and biofilm lifestyles which marked in black. The identified phosphoproteins were marked in red “P”. Venn diagram showed the amount of identified proteins from both lifestyles of *A. baumannii*.


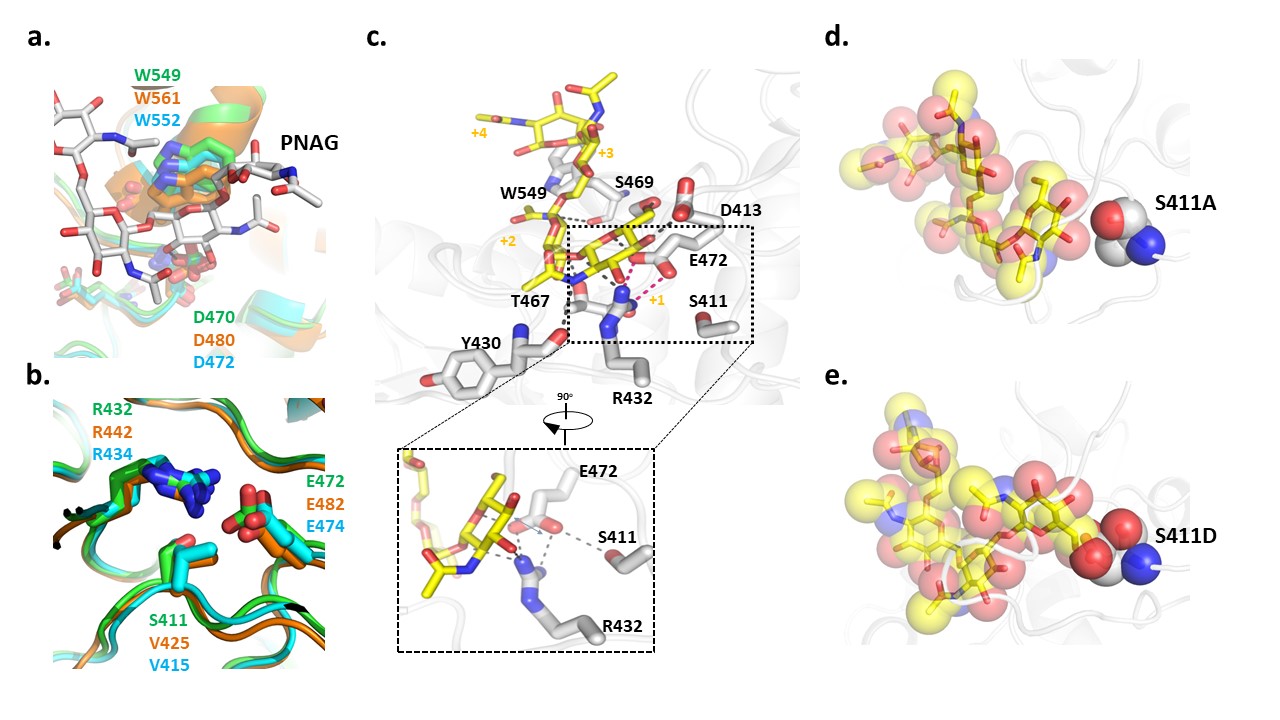


**Fig. S4. The AbPgaB1 modeling structure aligned with EcPgaB and BbPgaB with PNAG tetrasaccharide as a ligand.** (a) The structures of EcPgaB (PDB: 4p7r, cyan) and BbPgaB (PDB: 6au1, orange) were aligned with AbPgaB1308-659 (forest) with PNAG tetrasaccharide (grey). Residues W549 and D470 in AbPgaB1308-659 were highly conserved to BbPgaB (W561, D480) and EcPgaB (W552, D472). (b) Residues S411, R432, and E472 in AbPgaB1308-659 were highly conserved to BbPgaB (V425, R442, E482) and EcPgaB (V415, R434, E474) in this modeling structure. (c) The reducing end of GlcNAc tetrasaccharide (yellow) docked in AbPgaB1308-659 was defined as the +1 subunit. Residues R432 and E472 of AbPgaB1 participated in the strong coordination to +1 GlcNAc. The spatial arrangement of GlcNAc tetrasaccharide in AbPgaB1 when p-site Ser411 was replaced as Ala (d) or Asp (e).


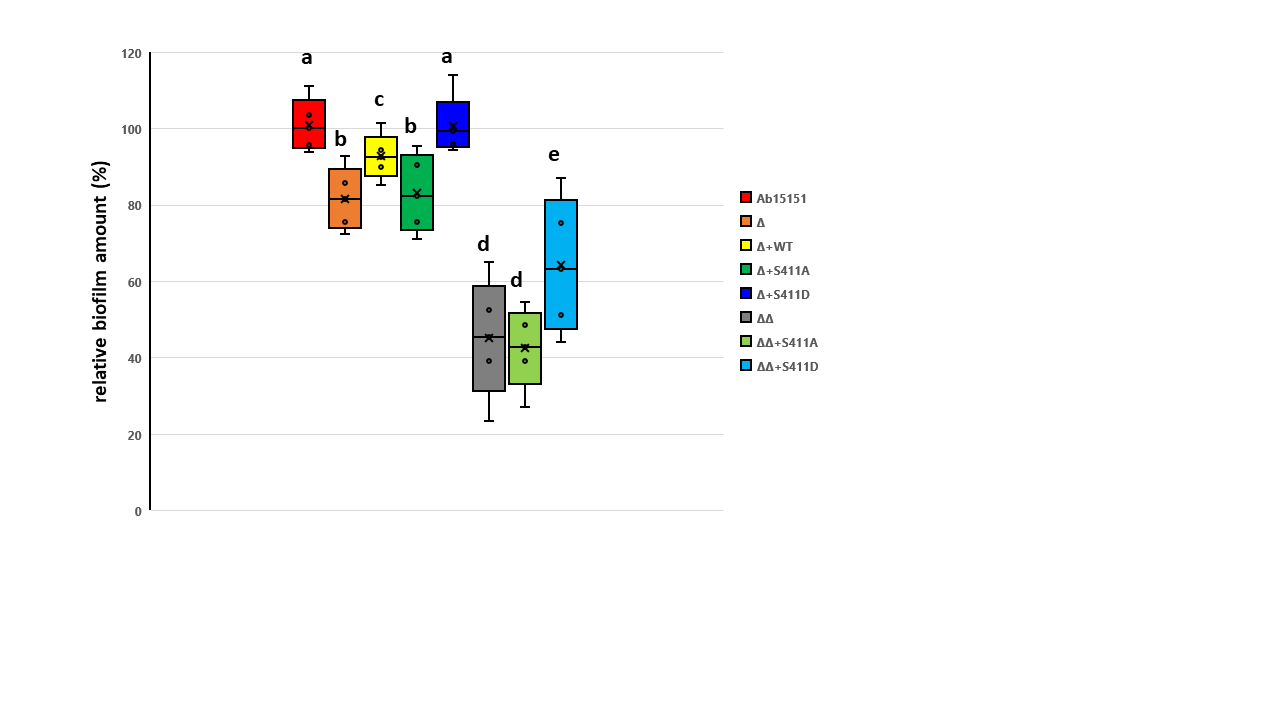


**Fig. S5. Biofilm quantification of *A. baumannii* WT and the PgaB-mediated deletion or complementation strains.** Biofilm was quantified by crystal violate staining and determined the absorbance at 595 nm. The OD_595_ values of Ab15151 were defined as 100% to calculate the relative biofilm amount from its derivative mutant strains. Each data point was averaged from at least 6 repeats. The PgaB1 deletion strain was abbreviated as Δ, while the PgaB1 and PgaB2 double deletion strain was abbreviated as ΔΔ. The complement expression of PgaB1 WT or its site-direct mutated derivatives were abbreviated as +WT, +S411A, or +S411D, respectively. The different labels of “a, b, c, d, or e” indicated the p-value of the t-test between each other was less than 0.001.


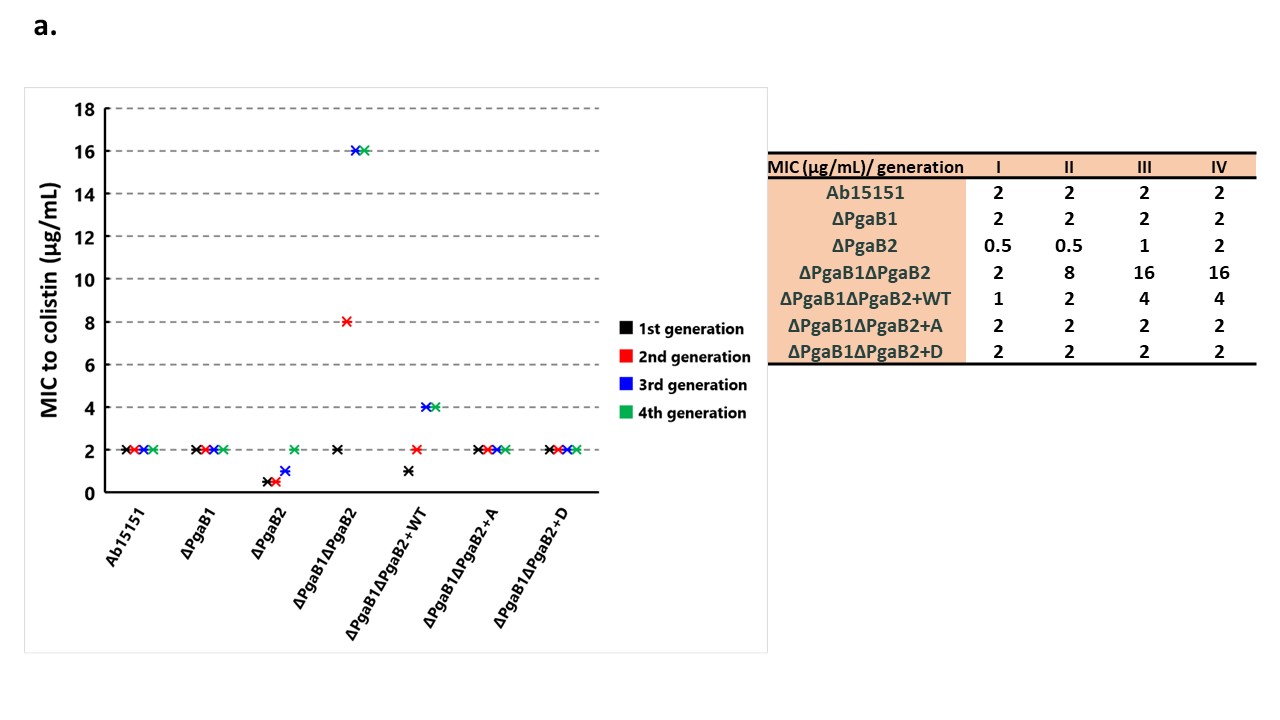


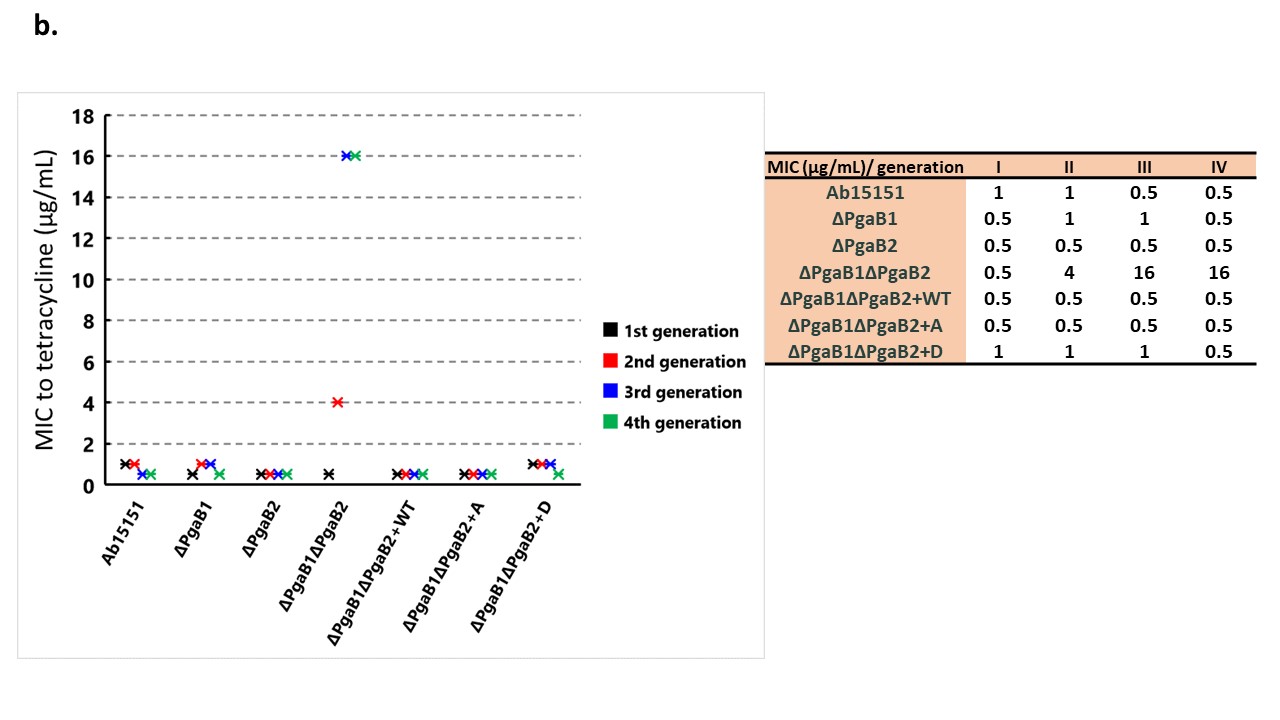


**Fig. S6. Minimal inhibition concentration of *A. baumannii* strains to colistin (a) and tetracycline (b) during 4 passages.** The MIC was determined by using the broth dilution method. The operon pgaBCD contained WT, S411A, or S411D mutated pgaB1 (+WT, +A, or +D) were constructed to complement the PgaB1 function in the ΔpgaB1ΔpgaB2 background. The inoculation of each *A. baumannii* strain from -80°C stock to LB medium and then incubated at 37°C with 250 rpm shaking for 16-24 hr was defined as the 1^st^ generation. The cultured bacteria broth was sub-cultured with a 1:1000 ratio to fresh medium and incubated under the same condition as 2^nd^ generation and so on. The sub-culture of Ab15151 and pgaB deletion strains did not stimulate with antibiotics while complement strains in this test need to supply 50 µg/mL kanamycin and 1.0 mM IPTG to express complemented PgaB1 and its derivatives.


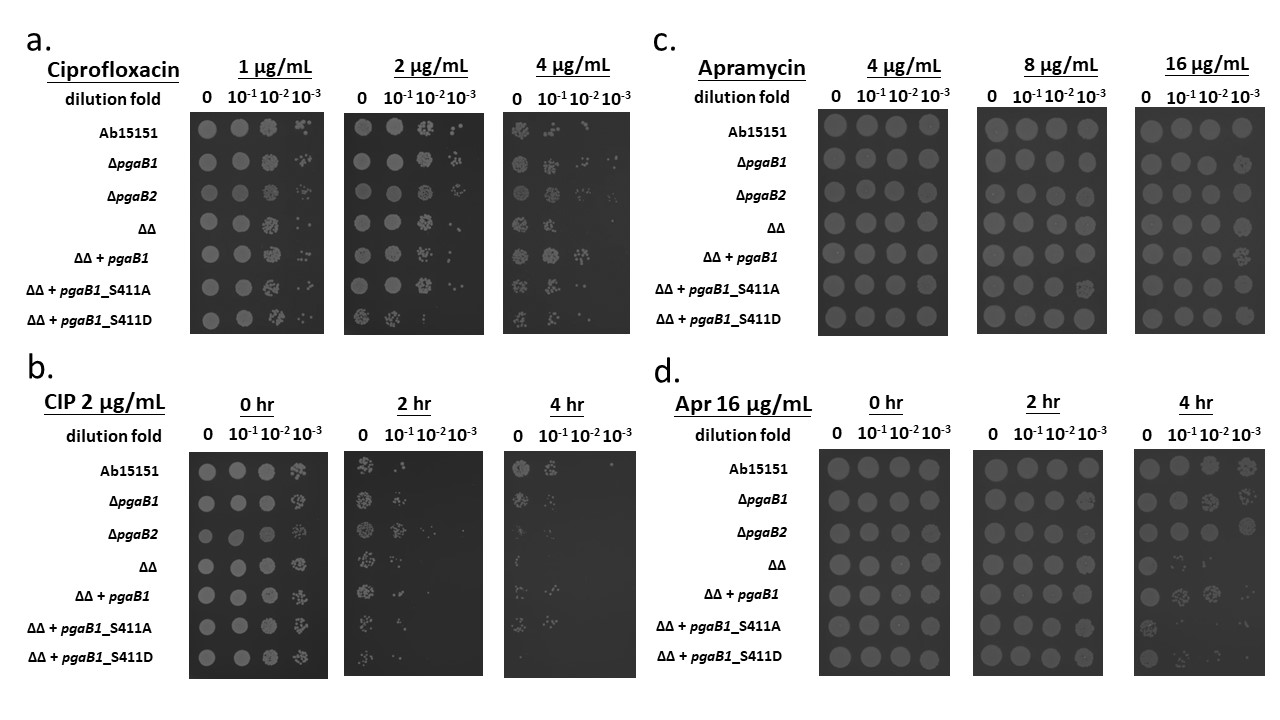


**Fig. S7. Time-dependent killing assay.** The overnight cultures were diluted to OD_600_ 0.01 for administration with antibiotic ciprofloxacin or apramycin. After 1 hr treatment with ciprofloxacin (a) or apramycin (c), the cultures were 10-fold serially diluted to spot on the LB agar plate. With the administration of 2 µg/mL ciprofloxacin (b) or 16 µg/mL apramycin (d) within 4 hr, all tested strains were 10-fold-diluted and incubated overnight for evaluating their survival rate.


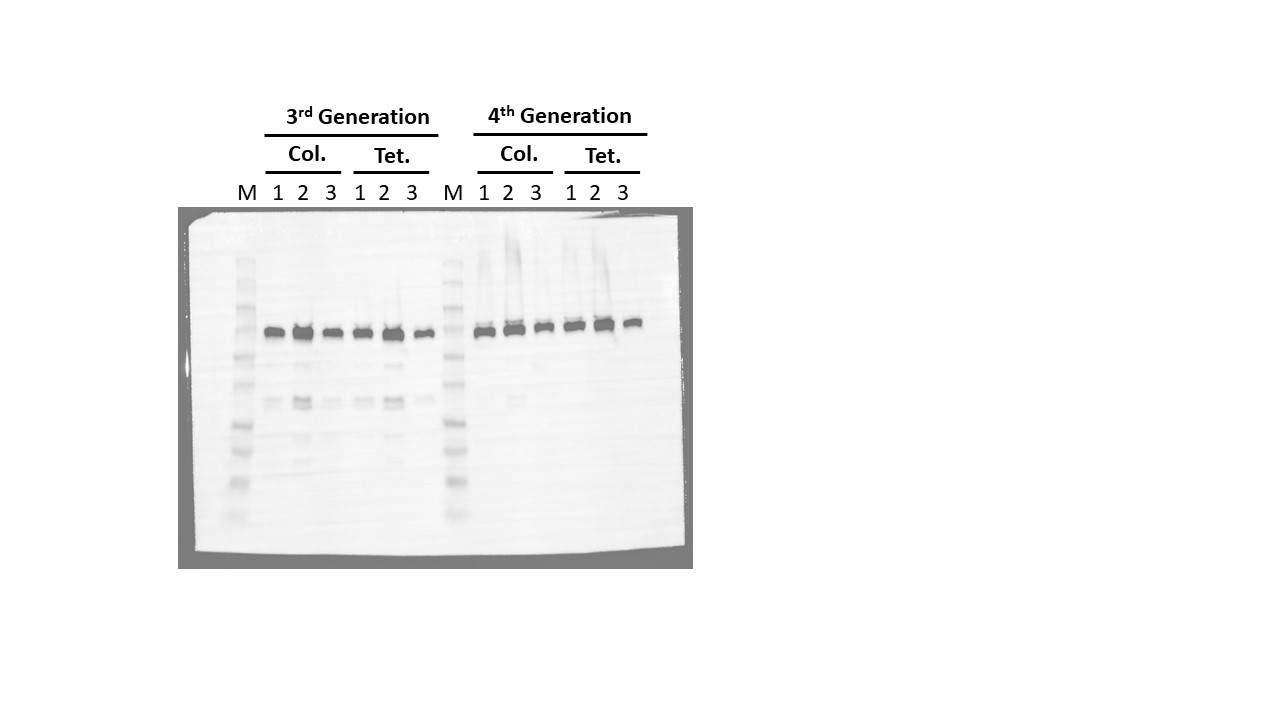


**Fig. S8. The complemented PgaB1 in PgaB deficient Ab strains were confirmed by Western blot.** To validate the effects of PgaB1 in MIC determination, the complemented PgaB1 (WT, S411A, or S411D) were checked by Western blot with anti-His antibody. The samples were harvested from the 3rd and 4th generations of MIC determination in Table 2 and Figure S5 when administrated with colistin (Col.) or tetracycline (Tet.). M, pre-stained protein marker; lanes 1 to 3 are ΔPgaB1ΔPgaB2 strain complemented with PgaB1 WT, S411A, or S411D, respectively. The molecular weight of PgaB1 fusion with 6-His tag was 70 KDa.

**Table S1. Identification of phosphorylated sites on AbPgaB1.**

| **Phospho-peptides** | **P-sites** | **Score** |
| --- | --- | --- |
| **EpHGIRSPR** | **H229** | **43.8** |
| **pTpDPVpSKpDLVVTEQAK** | **T407, D408, S411, D413** | **44.9** |
| **ApYAKQGLPTDLAK** | **Y482** | **50.9** |
| **WTApYKTK** | **Y507** | **41.4** |


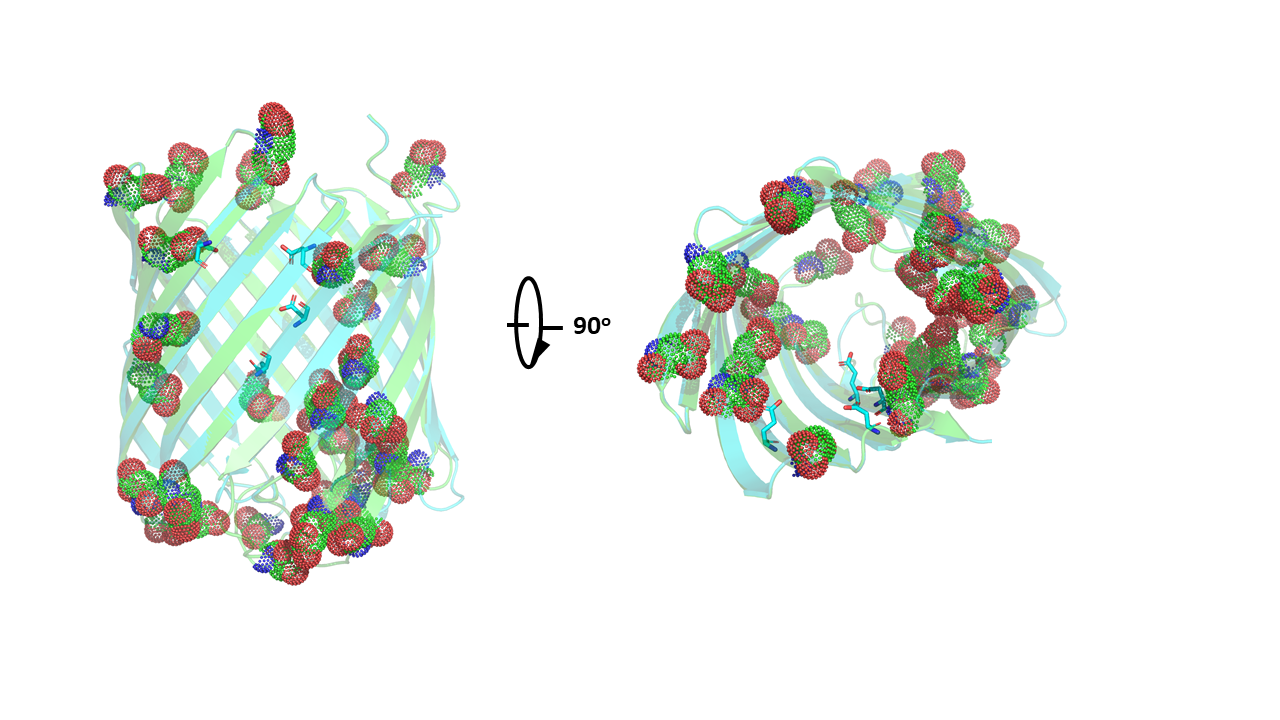


**Fig. S9. The predicted β-barrier structure of AbPgaA aligned with EcPgaA.** The structures of EcPgaA (PDB: 4y25, cyan) and AbPgaA (green) were aligned by PyMOL. The negatively charged residues E741, D777, E800, and D802 of EcPgaA were located in the porin structure which participated in the deacetylated PNAG binding preference. The negatively charged residues of the AbPgaA porin structure showed in dots. The left-hand side showed the side-view of the porin structure and the right-hand side showed the top view of the porin structure.
